# Realist review of low- to upper-middle-income country experiences on integration of HPV vaccination with other adolescent health services

**DOI:** 10.1016/j.vaccine.2025.126833

**Published:** 2025-03-19

**Authors:** Mary Carol Jennings, Sarah Nabia, Christopher Morgan, Chinelo Cynthia Nduka, Julia Brotherton, Megan Holloway, Katharine Bagshaw, Paul Bloem, Chizoba Wonodi

**Affiliations:** a Jhpiego, 1615 Thames St. #200, Baltimore, MD 21231, USA; b USAID's MOMENTUM Project, Country and Global Leadership, Washington, DC, USA; c Department of Community Medicine, Nnamdi Azikiwe University Teaching Hospital, Nnewi, Nigeria; d Melbourne School of Population and Global Health, University of Melbourne, Australia, Level 4, 207 Bouverie Street, University of Melbourne, Victoria 3010, Australia; e Independent Consultant. London, UK; f Public Health Institute, United States Agency for International Development's Global Health Training, Advisory, and Support Contract (GHTASC), Washington, DC 20024, USA; g World Health Organization, Av. Appia 20, 1202 Genève, Switzerland; h International Vaccine Access Center, Department of International Health, Johns Hopkins Bloomberg School of Public Health, Johns Hopkins University, 415 North Washington St, 5th Floor, Baltimore, MD 21231, USA

## Abstract

**Background:**

Integration of HPV vaccination with other adolescent health services may be associated with favorable programme outcomes including coverage, sustainability, and equitable delivery. Interest in such integrated approaches is building. However, the empirical evidence on how an integrated approach might be used to effectively deliver HPV vaccines and other adolescent health services has not been consolidated in a manner sufficient to inform policy and programmes.

**Methods:**

We conducted a realist synthesis review of published experiences from low- to upper-middle-income countries. Experts were invited as review commissioners, informing development and iteration of a programme theory of change (TOC). Our search strategy queried three large academic peer-review databases, grey literature, and a “snowball” of citations from key reference articles. We extracted learnings from specific real-world country experiences, exploring theoretical constructs describing the context in which an integration mechanism may deliver intended outcomes.

**Results:**

Of 1961 peer-reviewed articles and 16 grey literature articles identified, 8 articles met inclusion criteria, providing data from 6 country experiences to develop an iterated TOC. There were notable gaps in evidence regarding how an integrated approach might meet unique needs of underserved populations, including out of school youth and those affected by HIV. TOC iterations yielded 9 new factors and 4 discarded factors. Notable new factors included a multisectoral cooperation mechanism, linked to the use of a large number of other mechanisms, including integration of cost-sharing, supply chain distribution, and programme monitoring.

**Conclusion:**

This realist synthesis is to our knowledge the first review consolidating operational evidence on how an integrated approach might be used to effectively deliver HPV vaccines and other adolescent health services. Our iterated TOC provides a framework to guide future implementation research, to ensure attribution of observed effects to an integrated approach is merited – and to inform decision-making in adolescent immunization policy and programmes.

## Highlights

• Realist synthesis articulates theory of change for integrated immunization service delivery.
• Multi-sectoral cooperation in planning for integrated services is an important mechanism.
• Evidence heat map illustrates research gaps in factors affecting adolescent health services.

## 1 Introduction

In 2020, the World Health Assembly adopted a global strategy to accelerate the elimination of cervical cancer as a public health problem, aiming to shift the needle on one of the most common cancers and causes of cancer-related death in women across the globe, through three pillars: vaccination, screening, and treatment, setting specific scale-up targets for each by 2030 [1]. However, progress toward these targets has been uneven, particularly in low- and lower-middle-income countries (LMICs), where women experience 84 % of the ∼660,000 new annual cases and between 87 and 90 % of the annual 350,000 deaths - incidence that is double and death rates that are roughly triple those of high-income countries (2022 data) [2,3]. Along with low rates of screening and treatment, HPV vaccination coverage remains low, at 10 % across the 57 Gavi-supported countries in 2022 [4].

Varied stakeholders have proposed that integration of HPV vaccination into Primary Health Care (PHC) and routine vaccination programs may enhance HPV vaccine coverage, programme sustainability [5], and ability to support uptake among local priority populations such as out-of-school girls, migratory or conflict-affected populations, or youth living with HIV [6], while harnessing new resources to better equip health systems to meet the underserved needs of adolescent populations [7,8]. Although primary health care services available for adolescents span a wide range, from nutrition and vision screening to age-appropriate sexual and reproductive health screening and education [9], a common factor across countries is low and inconsistent uptake [10]. A number of low- to upper-middle-income countries have piloted [11] or undertaken the integration of HPV vaccine into existing adolescent health programs, or have undertaken the integration of other adolescent health services into HPV vaccine delivery efforts [[12], [13], [14], [15]]. However, the theoretical constructs describing why an integrated service delivery approach may contribute to intended outcomes, in which contexts, and for which populations, have not been fully explored; there have been limited investments; and current policy guidance lacks implementation evidence [8].

As countries develop strategies to revitalize their national HPV vaccine service delivery, an action-oriented synthesis of operational evidence is urgently needed to articulate how and why integrated approaches might strengthen both adolescent health programs as well as HPV vaccine programs, now and into the future, and how integration may facilitate pro-equity and sustainable programs and health systems.

To address this gap in data to guide policy and programs on integrated service delivery for HPV vaccination, we conducted a realist synthesis review to address two specific implementation research questions:

1. Through which mechanisms is HPV vaccination being effectively integrated with other adolescent health services?
2. What contextual factors show evidence of contributing to the outcomes documented for integrated approaches?

## 2 Methods

A realist synthesis methodology is uniquely appropriate to generate an action-oriented synthesis of a complex body of data [16], and the method places particular emphasis on understanding causal pathways [17]. We selected this methodology and approach to develop a better understanding of how and why integrated service delivery may lead to desired programmatic outcomes for HPV vaccine programs and adolescent health services.

To ground our realist synthesis approach in practical theory and to align with common realist practice [18,19], our review team identified and invited a selected group of stakeholders who are experts in the field of HPV vaccine implementation and cervical cancer prevention, drawing representatives from the Coalition to strengthen the HPV Immunization Community (CHIC) [20], US Agency for International Development (USAID), Gavi and WHO, to serve as review commissioners. Following the realist synthesis approach, we worked with these commissioners to develop and iterate a programme theory of change (TOC) that articulates the specific *context*, such as service delivery setting, in which a particular *mechanism* of change, such as co-delivery of deworming medications alongside HPV vaccination, is associated with a relevant *outcome* – such as a change in awareness or uptake of services [17]. Commissioners supported formulation of research questions; advised on research methods; and responded to findings of the review in an ongoing manner. This shaped interpretation and synthesis of results, and guided testing of the TOC, aiming to discern where implementation evidence may be sufficient to translate into action, where there are gaps, and how to evolve the programme TOC – all intended to deepen understanding of how context and causal processes (“mechanisms”) interact to contribute to an outcome [21].

With commissioner input, we developed an initial search strategy to query peer reviewed databases, to select a small number of representative “reference” articles, and to assess relevant grey literature sources. Commissioners submitted lists and websites with potential grey literature to the review team, including programme evaluations, policy documents, and reports from HPV vaccine integration experiences. We worked with an academic library informationist to develop a search strategy of MeSH terms and Boolean operators for PubMed, Embase, and Scopus databases (Appendix I). We validated our search findings and used a “snowball” approach to search reference lists of retrieved articles and to screen bibliographies of a short list of relevant publications [5,7,9,15] identified by commissioners. We managed screening workflow for peer reviewed articles with Covidence, a web-based collaboration software platform that streamlines literature review processes [22]. We ran the peer-reviewed database query March 03, 2022 and implemented the full search strategy between March 2022 and September 2022.

We included studies that:

• documented an intervention involving the integration of HPV vaccines with other adolescent health services;
• delivered services to an adolescent or youth population;
• measured a programme outcome (examples included: feasibility, acceptability, cost, coverage, uptake);
• were original research; and
• were considered of admissible quality.

Review articles, studies involving only adults, studies reporting work conducted only in high-income countries as per World Bank's 2021 categories, and non-human research studies were excluded.

We assessed these criteria in the initial screening phase through a list of 9 questions, refining the question list after piloting, and using a similar, refined set of questions for article selection and final appraisal for data extraction.

We developed a data abstraction and analysis matrix to identify key context, mechanism and outcome factors from each article. Commissioners provided input and discussion to guide the definition of these terms and the factors that we extracted, throughout the process of extraction and during initial analysis. Two reviewers independently extracted data from each paper. A third reviewer arbitrated the reconciliation of differences in data extracted.

In order to assess study quality, we classified each article by its design, according to the validated Cochrane Effective Practice and Organization of Care group (EPOC) criteria [23]. We then assessed quality using a modified GRADE [24] and CASP-Qualitative study quality assessment tool [25], according to study design classification. Included studies were those that passed general quality and general acceptability screening for these tools.

We mapped extracted evidence into our initial programme TOC (supplemental files), iterating with inputs from each article's specific country experience to provide insight into potential relationships between the context, integration mechanism, and associated outcomes. In order to provide useful guidance to efforts to further develop the research base on this topic, we also mapped availability and absence of evidence onto our theory of change.

## 3 Results

**Fig. 1:**
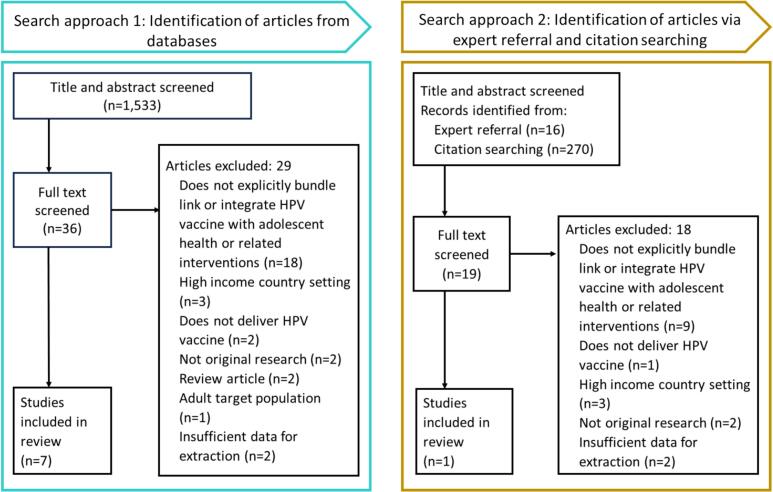
Document flow diagram.

Articles via Expert Referral and Citation Searching:

Fifteen grey literature articles were identified at the outset of the grey literature search through expert referral, through an active process conducted by our commissioners and authors; one additional article was identified in iterative searching (Fig. 1). All sixteen underwent full text review. All but one [26] were excluded, with reasons for exclusion presented in Fig. 1. Two hundred and seventy titles and abstracts were identified from the reference lists of the four selected relevant articles (listed above). Of these only three articles were not captured in the database search (below) and met criteria to undergo full text screening. None of these three progressed to data extraction because they were either not focused on HPV vaccine delivery (*n* = 1), not original research (n = 1), or there was insufficient data for extraction (n = 1).

Articles from Databases:

Our search of peer-reviewed academic databases yielded 1,961 articles. We imported these into Covidence for screening, removing 428 duplicates. Two reviewers independently screened title and abstracts against inclusion criteria for the resulting 1,533 records, excluding 1,497. A third reviewer arbitrated discrepancies in screening determination. Two reviewers independently reviewed the full text of the resulting 36 articles, excluding 28 with reasons listed in Fig. 1. Three of the initially resulting 11 articles were excluded during the final iteration of data extraction and analysis. Of these, one was a prospective non-controlled case study in Brazil which did not explicitly bundle or link HPV vaccine with other services [27]; another was a qualitative programme assessment of lessons learned after HPV vaccine introduction in four unnamed countries, one of which briefly described an integration approach but in insufficient detail for data extraction [28]; and one was a cross-sectional qualitative article, a lessons learned programme description of the national roll-out of South Africa's HPV vaccine programme that integrated other health education topics with vaccine delivery, which provided too few data on integrated services for extraction [14]. These were assessed to be low quality/generalizability, medium quality/generalizability, and low quality/generalizability, respectively.

Eight articles were assessed as suitable for data abstraction and inclusion in the final analysis (Table 1). Two of the final included studies were ascertained to be of good [29] or high [26] quality; the remaining six studies were of low or very low [30] quality and generalizability. Despite the varying quality and rigor of the articles, the research team and commissioners came to consensus that all eight of the final studies selected for inclusion provided meaningful inputs to test against our TOC.

**Table 1:** Overview of Included Articles.

References	Country	Country Income Category for 2021	Scale and Duration of Intervention	Brief description of intervention	Target Population
LaMontagne 2011 [31]; Mugisha 2015 [32]	Uganda	Low-income country	1 District, over 1 year, 2008–2009	Demonstration project used age-based eligibility strategy to integrate delivery of HPV vaccine with deworming medications, Vitamin A supplements, and catch-up of other routine immunizations, through an existing school-based twice-yearly campaign style Child Days Plus program.	Girls aged 10 (2263 eligible girls enumerated)
Binagwaho 2012 [33]	Rwanda	Low-income country	National, over 1 year, 2011–2012	National introduction used grade-based eligibility strategy, integrating delivery of HPV vaccine with health education on hygiene, nutrition, infectious diseases and reproductive health, through a 6-day national “health days” campaign of service delivery in schools and then in communities, with CHW tracking and referral to facility, for girls not in school.	Girls in primary school grade six; girls aged 12 if out-of-school
Moodley 2013 [34]	South Africa	Upper-middle-income country	1 District, less than 1 year, 2011	Demonstration project implemented the newly revised National School Health Policy and Implementation Guidelines at the District level, integrating HPV vaccine into a comprehensive, integrated school health programme as part of a primary healthcare package, using existing school health nurses and infrastructure to co-deliver HPV vaccine with other services (clinical appraisal and collection of temperature, height, weight, arm circumference), and ensure completion of vaccine schedule.	Girls aged 9–12 and/or girls in grade 4 or 5 (both criteria used).
Buang 2018 [29]; Muhamad 2018 [30]	Malaysia	Upper-middle-income country	National, over 7 years, 2010–2017	National introduction first used age-based, then a grade-based, eligibility strategy to integrate delivery of HPV vaccine into the existing school health program, which remained responsible for developmental assessments, health screening/education, and booster vaccinations of other routine immunizations (measles, rubella, diptheria, tetanus toxoid) throughout the year. Services provided for students from preschool through age 16, but HPV vaccine was the only service provided to age 13 / Form 1.	Girls aged 13 at outset; school-based grade cohort approach starting in in 2016. No discussion of strategy for girls not in school.
Engel 2022 [11]	Togo	Low-income country	2 Districts, over 2 years, 2016–2017	Demonstration project used age-based eligibility approach to integrate an age-appropriate health education curriculum (puberty education, menstrual hygiene, sexual and reproductive health and rights, prevention of early pregnancy and STIs, and hand-washing practices) with delivery of HPV vaccine through a once-yearly, campaign-style, school-based delivery model.	Girls aged 10 were eligible for HPV vaccine. (15,272 girls enumerated for 2nd dose). Boys and girls aged 9–13 were eligible to receive the educational curriculum component.
Morgan 2022 [26]	Tanzania	Lower-middle-income country	6 District Councils in 1 Region, over 2 years, 2019–2021	Government and its partners consulted communities to design the “HPV-Plus” intervention for initial national HPV vaccine introduction, which used existing health-facility-based vaccinators to co-deliver HPV vaccine with a package of age-tailored adolescent health services. Vaccinators were responsible for the schools within their local catchment area. Integrated services included an educational curriculum on adolescent health, sexual and reproductive health, and HPV and cervical cancer. Girls were also screened for nutrition and vision problems and offered antiparasitic (deworming) medication. Girls affected by HIV received supplemental HPV vaccine dose.	The intervention differed by gender and age: Only girls aged 14 were vaccinated; the educational component was offered to girls and boys aged 10–14 years; vaccination and screening was offered to girls aged 14; 118,000+ girls and boys were reached over 14 months.

### 3.1 Overview of included articles

The eight included articles were conducted across six countries, five in sub-Saharan Africa (Uganda – 2, Togo – 1, South Africa – 1, Rwanda – 1, Tanzania – 1) and one in Southeast Asia (Malaysia – 2) (Table 1). Six were observational programme evaluations (*n* = 6), one was a cross-sectional study, and one was a qualitative study. Implementation described in the articles spanned 13 years, from 2008 through 2021. Articles reported on study or programme evaluation periods ranging from 1 to 7 years, with a median of 1.5 years.

### 3.2 Cross-cutting findings and iteration of theory of change

**Fig. 2:**
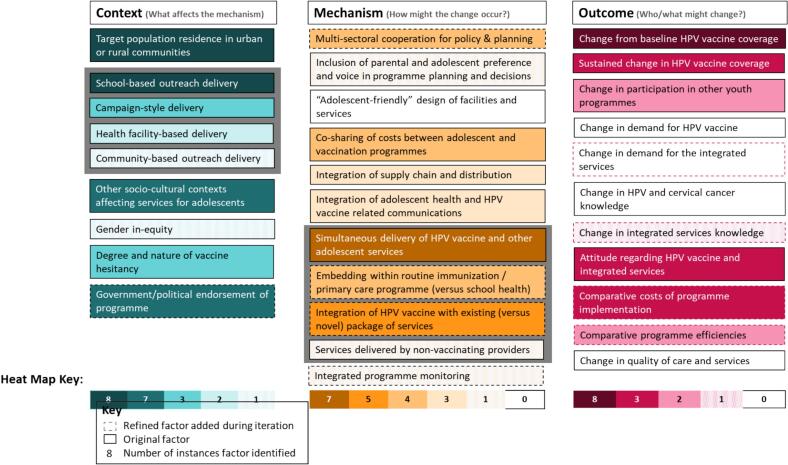
Iterated Theory of Change with Heat Map of Possible Context, Mechanism, and Outcome Factors Active in an Integrated Approach for HPV Vaccination.

We synthesize findings as presented in our iterated TOC (Fig. 2), followed by discussion of context-mechanism-outcomes (C-M—O) configurations and learnings from each of the six countries.

### 3.3 Evidence on ‘Context’

Included articles provided evidence for each of the seven ‘context’ factors postulated in the initial TOC, and iteration supported the addition of one new factor (Fig. 2). The most frequently reported context for an integrated approach was school-based outreach delivery, presented in all eight articles. Three articles assessed integrated service delivery within a vaccination campaign [11,32,33] (Uganda, Rwanda, Togo). Two articles briefly described the use of a facility-based approach, without specifying if integrated services were available [32,33] (Uganda and Rwanda). One article reported on an integrated community-based outreach approach [32] (Uganda). One article assessed gender inequity [26] (Tanzania), and seven articles assessed other socio-cultural contexts affecting services for adolescents, such as religion, high HIV prevalence, or rates of school enrollment [26,[29], [30], [31], [32], [33], [34]] (South Africa, Malaysia, Rwanda, Tanzania, Uganda). TOC iterations differentiated these factors. Three articles documented context challenges to vaccine confidence, reporting on rumors, myths, and steps taken to reduce vaccine hesitancy [29,30,32] (Malaysia, Uganda). One new context factor emerged through TOC iteration: seven articles reported on the contextual role of government and/or political endorsement of an integrated programme approach, either through official communications of government support [11,32,34] as in South Africa, Uganda and Togo, or in relation to an emergent mechanism factor (below), of intentional multisectoral cooperation [26,29,30,33] in Malaysia, Tanzania, and Rwanda.

### 3.4 Evidence on ‘Mechanism’

Included articles provided evidence for six of the eight mechanisms postulated in the initial TOC, and four mechanisms were added through TOC iteration (Fig. 2). Four articles described multisectoral cooperation for policy and planning [26,29,30,33] in Malaysia, Rwanda, and Tanzania; TOC iterations adopted this mechanism. One article reported the use of an integrative mechanism involving explicit inclusion of parental and adolescent preference to design an integrated service delivery package and program [26] in Tanzania; we retained this factor in TOC iterations. We found no evidence of “adolescent friendly” facilities; however, we reached consensus to retain this factor in TOC iteration. The initial TOC postulated both a cost-sharing and a decreased delivery cost mechanism. Four articles reported cost sharing between adolescent health and vaccination programs [26,[30], [31], [32],^34^] in Malaysia, South Africa, Tanzania and Uganda, but TOC iterations captured changes in delivery cost as an outcome (see below). Three articles reported integration of supply chain and distribution [26,29,32], in Malaysia, Tanzania, and Uganda; TOC iterations retained this factor. Three articles described a mechanism of integrating communications between adolescent health and HPV vaccination programs [11,26,33], in Rwanda, Tanzania and Togo; TOC iterations retained this factor. The most frequently reported mechanism of integration was simultaneous delivery of HPV vaccine and other services, presented in seven articles [11,26,29,[31], [32], [33], [34]] (all six countries) and referenced indirectly in the eighth [30]. TOC iterations parsed novelty of the integrated package from staffing concepts. All countries used existing school health or routine immunization staff, with one article reporting the use of non-vaccinating healthcare providers to deliver the integrated services package [34] (South Africa). The TOC was refined to include a new factor, on whether an integrated approach used a novel [11,26,33,34] (Rwanda, South Africa, Tanzania, Togo), or an existing package [[29], [30], [31], [32], [33]] (Malaysia, also Rwanda, and Uganda) of other adolescent health services. Further TOC iterations yielded an additional context factor – which system ‘hosted’ the integrated services. Three countries integrated services into the routine immunization or primary health care programme (Rwanda, Tanzania and Uganda) [26,[31], [32], [33]], and four integrated into the school health programme (Malaysia, South Africa and Togo) [11,29,30,34]. A grey TOC box indicates this close relationship (Fig. 2). Finally, one article (Malaysia) reported the integration of programme monitoring [29]; TOC iterations adopted this factor.

### 3.5 Evidence on ‘Outcomes’

Nine ‘outcome’ factors were postulated in the initial TOC, including four metrics intended to capture nuances of coverage. Through TOC iteration, we discarded two and retained four outcome factors, while adopting four new factors (Fig. 2), effectively condensing coverage metrics. A “change from baseline HPV vaccine coverage” was measured in all eight articles [11,26,[29], [30], [31], [32], [33], [34]], largely describing initial coverage in new programs, while a “sustained change in HPV vaccine coverage” metric measured in three articles captured coverage over a longer time course, over 7 years in Malaysia and 14 months in Tanzania [26,29,30]. There was no evidence of measurement of adolescent health-seeking behaviors (from initial TOC), but two articles did report on change in participation in integrated programs (also in initial TOC), in Tanzania and Togo [11,26]; this was captured in TOC revisions. We also saw no evidence of the HPV vaccine ‘demand’ outcome from the initial TOC, but we reached consensus to retain the concept in TOC iteration – acknowledging that ‘demand’ and ‘health seeking’ concepts overlap. Similarly, we found no evidence of HPV and cervical cancer knowledge outcomes from the initial TOC, although one article reported on change in knowledge of the integrated services, in Togo [11]. The refined TOC presents these as two separate knowledge measures. Three articles reported on a change in attitudes [11,26,30], capturing attitudes about the combined, integrated services (acceptance, parent willingness to consent), rather than an attitude unique to the vaccine or other services, in Tanzania, Togo and Malaysia. TOC iterations present a combined “attitude” factor. TOC iterations adopted the outcome of costs of integrated service delivery, reported in Malaysia, Tanzania, and Uganda [26,29,32]. TOC iterations also adopted a programme efficiency outcome (stock-outs, school disruption, from Tanzania and Uganda) [26,32]. No articles presented data on quality of care or services, however, we reached consensus to retain this concept in TOC iterations. Similarly, we found no use of a zero dose outcome; TOC iterations discarded this concept, with Commissioners noting its decreasing importance as countries adopt a single-dose schedule.

### 3.6 Malaysia

In 2010, Malaysia incorporated its new national HPV vaccine program into the existing school health programme (Table 2), which had a strong track record delivering other vaccines (measles/rubella and diptheria/tetanus toxoid boosters) to younger students (age 7) [29]. Both included articles reported on the socio-cultural context of religiously influenced beliefs held across the country's sizeable Islamic population, describing the intentional use of culturally tailored demand generation messaging ahead of introduction [29,30]. Malaysia used multiple mechanisms for an integrated approach: HPV vaccine was delivered simultaneously alongside other existing adolescent health services, including other vaccines, embedded into the school health program; the package was delivered by vaccinators; staffing costs were shared between the school health and vaccination programs; and the country integrated budgetary processes, supply chain logistics and distribution programmes, and programme monitoring workflows and infrastructure [29]. Malaysia also explicitly used a mechanism of multi-sectoral cooperation for policy and planning, discussed by one included article as a key factor underpinning success of the integrated approach [29], and apparent in programme planning, communications, and monitoring. Neither article assessed explicit relationships between these mechanisms and reported outcomes, but both reported an increase in population coverage and number of girls vaccinated, along with a sustained level of high coverage, and a sustained change in attitude (parental consent and acceptance) (Table 2). One article reported generally on comparative programme costs, linking multisectoral coordination to the concept of reducing costs in the author's discussion [29]. The Malaysian example, which continues today, particularly supports the addition of the multi-sectoral collaboration mechanism to our iterated TOC.

**Table 2:** Context-Mechanism-Outcome Configurations from Country Experiences with Integrating HPV Vaccine and Other Adolescent Health Interventions.

Reference(s)	Country	Context	Mechanism	Outcome	Limitations
LaMontagne 2011 [31]; Mugisha 2015 [32]	Uganda	• School-based delivery • HPV vaccine was integrated into community-based delivery of existing ‘Child Days Plus’ programme (deworming medicines and Vitamin A) [32] • Integrated programme in 1 district, Nakasongola (2263 girls) [31,32] • Age-based (10y/o) selection criteria for integrated package [31,32] • Out-of-school girls referred to health facilities or reached through scheduled outreach sessions for other vaccines [32] • Rural & urban programme settings • High cervical cancer burden, good local EPI performance, politically stable [32] • Government / political endorsement improved vaccine acceptability: created trust among health & education personnel [32]	• **Simultaneous delivery of HPV & other services**^1^: HPV vaccine + ‘Child Days Plus program’ [31,32] • **Co-sharing of costs between adolescent and vaccination progammes**: Package delivered by existing routine immunization programme staff with focus on shared budget [31,32] **• Integration of supply chain and distribution**: HPV transport and distribution concurrent with routine vaccines [32] **• Embedding within routine immunization / primary care programme** [32], with **• Integration of HPV vaccine with existing package of services** [31,32]	**• Change from baseline HPV vaccine coverage**: Programme area with baseline of zero reached 60.7 % endline coverage over one year period (2009) using administrative data [31,32]. • **Comparative programme efficiencies**: School disruption reported as higher in age-based, service integrated approach versus grade-based with no integration [32].	Age-based integrated service delivery approach was compared to grade-based non-integrated approach; differences in coverage cannot be attributed to the integration component. Caregiver reasons for non-vaccination included lack of awareness of the programme and difficulty determining eligibility by age.
Binagwaho 2012 [33]	Rwanda	• School-based, campaign-style delivery • National-level programme was the first low-income country HPV vaccine introduction globally • Grade-based (grade 6) selection criteria • Out-of-school girls reached through age-based (12y/o) campaign efforts • Rural & urban programme settings • Sensitization campaign included training of teachers • Strong support of national politicians ahead of introduction	**• Multi-sectoral cooperation for policy & planning**: Integration was overseen by both Ministry of Health and Ministry of Education. NITAG added new technical working groups on education, HIV/AIDS, gender/family promotion, cancer, to coordinate planning, programme rollout and evaluation (cold chain, procurement, enumeration, data collection) **• Integration of adolescent health and HPV vaccine related communications**: HPV vaccine delivered by local health care providers and teachers on educational “Health Days” **• Simultaneous delivery of HPV vaccine and other adolescent services**: teachers and local health care professionals collaborated to deliver package **• Embedding within routine immunization / primary care programme** **• Integration of HPV vaccine with novel *and* existing package components**: Project created “Health Days”, adding to existing educational curriculum and delivering vaccine (hygiene, nutrition, infectious diseases, reproductive health)	**• Change from baseline HPV vaccine coverage**: Programme area with baseline of zero increased to 93.23 % in a final programme evaluation.	Integration was an explicit part of the intervention, but not the focus of the programme evaluation; minimal data was available. Timing of 3 rounds of evaluations unclear.
Moodley 2013 [34]	South Africa	• School-based delivery • Integrated programme in one district, Zululand, within KwaZulu Natal province • Both grade-based (grade 4 or 5) and age-based (9-12y/o) selection criteria • Rural & urban programme settings • District characterized by widespread poverty, high HIV incidence, poor access to basic services & facilities • Government created a national policy to support addition of HPV vaccine into existing school-based health program	• **Co-sharing of costs between adolescent and vaccination progammes**: through staffing budget efficiencies • **Simultaneous delivery of HPV & other services**: General clinical and nutritional appraisal (measuring temperature, height, weight, mid arm circumference) selected to be delivered at same time as vaccination • **Embedding within school health programme**: Package delivered through existing school-based primary health care programme by school health staff • **Integration of HPV vaccine with novel package of services** described in new policy • **Other services delivered by non vaccinators**: School nurses learned to administer HPV vaccine and conducted clinical appraisal	**• Change from baseline HPV vaccine coverage**: Reported increase in number/proportion of enumerated adolescent girls vaccinated, from zero at baseline to 97.8 % in final programme evaluation.	Integration not an explicit focus of the programme evaluation.
Buang 2018 [29]; Muhamad 2018 [30]	Malaysia	• School-based delivery [29,30] • Nation-wide programme [29,30] • Grade-based (grade 7) selection criteria [30] • Rural & urban programme settings • National school health programme had a strong track record providing measles/rubella and diptheria/tetanus toxoid vaccination • Immunization programme worked with national Islamic religious authority (JAKIM) to issue statement (fatwa) on the religious alignment with HPV vaccination [29,30] • Addressing vaccine skepticism: organized public fora, media campaigns, survey among teachers & school health teams to identify doubts & address then accordingly [29,30]	• **Multi-sectoral cooperation for policy & planning**: Formal collaboration between Ministry of Education & Health on demand generation & communication strategy [29] • **Simultaneous delivery of HPV & other adolescent services**: HPV vaccine integrated into existing programme of regular development assessment, booster vaccinations & health education [29] • **Embedding within school health programme** [29,30] • **Co-sharing of costs between adolescent and vaccination progammes**: Ministry of Health nurses mobilized to deliver package along with school health teams [30] **• Integration of supply chain and distribution:** HPV procured through regular budgetary allocation, usual logistics & cold chain [29] • **Integration of HPV vaccine with existing package of services** [29,30] • **Integrated programme monitoring**: HPV monitoring integrated into ongoing school health program at district & state levels [29]	**• Change from baseline HPV vaccine coverage**: Programme area with baseline of zero increased to 82.6 % (2016) in a final programme evaluation [29,30]. **• Sustained change in HPV vaccine coverage**: Among annual target population of 250,000 girls, programme reached 227,834 girls in 2010 and maintained similar levels at 213,187 in 2016 with 3rd dose HPV [29,30], i.e. maintained coverage at 84–85 % through 2016 [29,30]. **• Change in attitude regarding HPV vaccine**: vaccine acceptance (parental consent) increased from 95.9 % (2010) to 98 % (2016) [30].	Effect of integration not assessed explicitly.
Engel 2022 [11]	Togo	• Once-yearly school-based campaign-style delivery • 2 districts – Golfe and Tchamba • Age-based selection criteria: 10y/o girls for HPV vaccination and 9 to 13y/o boys for health education. • Boys explicitly included, addressing gender inequity topics • Special outreach strategy for out-of-school girls. • One rural & one urban programme setting	• **Multi-sectoral cooperation for policy & planning**: Ministry of Health and Education jointly engaged in planning and delivering the age-appropriate adolescent health educational curriculum on puberty education/menstrual hygiene and hand-washing, and vaccination services • **Integration of adolescent health and HPV vaccine related communications**: In Golfe, health care workers provided both vaccination and educational curriculum; in Tchamba the curriculum was provided by teachers while health care workers administered vaccines **• Embedding within school health programme** • **Integration of HPV vaccine with novel package of services** selected to improve adolescent well-being	**• Change from baseline HPV vaccine coverage**: Programme area with baseline of zero (2015) increased to 91 % (2017). **• Attitude regarding HPV vaccine and integrated services**: HPV vaccine in conjunction with educational health curriculum was well received by health care workers, teachers, parents. **• Change in participation in other youth programs**: 93 % teachers confirmed all students participated in educational curriculum sessions. **• Change in integrated services knowledge**: teachers reported 3 in 4 adolescents knew/recalled educational curriculum messages.	Article describes a demonstration project that specifically aimed to assess integration feasibility. Feasibility was demonstrated, however, intervention was not subsequently scaled up to the national level with the HPV vaccine program. Discussion of inputs to this decision were beyond scope of article.
Morgan 2022 [26]	Tanzania	• School-based delivery • Some community-based outreach delivery for out-of-school girls • Context of HIV/AIDS burden included in programme planning • 6 sub-national, District Councils, across Njombe region • Age-based selection criteria for target population: 10 to 14y/o boys and girls for health education, 14 y/o girls for HPV vaccination & other health services • Gender inequity addressed by offering educational curriculum components to boys and girls • Rural & urban programme settings. • Government created a national policy to support integrated HPV vaccine and adolescent health service delivery, aiming to strengthen adolescent health services	• **Multi-sectoral cooperation for policy & planning**: planning and monitoring activities integrated into broader primary health care and vaccination meetings; teachers and educational workers involved in integrated service planning and delivery • **Inclusion of parental and adolescent preferences in programme planning and decisions**: participatory, human-centered design approach used to develop the programme and inform selection of integrated service delivery package **• Co-sharing of costs between adolescent and vaccination programmes**: primarily through staffing mechanisms **• Integration of supply chain and distribution**: adding HPV vaccine and leveraging existing system for package commodities **• Integration of adolescent health and HPV vaccine related communication**: across school health system, primary health care and routine immunization system **• Simultaneous delivery of HPV vaccine and other services**: HPV-Plus package provided vaccine with screening for nutrition, vision problems, nutritional advice, and deworming **• Embedding within routine immunization programme**: delivered by routine immunization workers • **Integration of HPV vaccine with novel package of services** selected with community input	• **Change from baseline HPV vaccine coverage**: Programme area with baseline of zero increased to 80 %. • **Sustained change in HPV vaccine coverage**: HPV vaccination activity and coverage was maintained in the programme area over the full 14 months of the study. • **Change in participation in other youth programs**: 15,920 adolescent boys & girls reached with HPV-Plus educational curriculum. In total, the programme reached 118,000 boys & girls between 10 to 14y/o **• Attitude regarding HPV vaccine and integrated services**: Positive attitudes (acceptability) reported for integrated approach. • **Comparative costs of programme implementation**: Costs were reported but detailed comparison not performed. • **Comparative programme efficiencies**: deworming medications experienced stockouts. Staffing constraints also noted.	Difficulties noted in reaching adolescents not in school. Deworming medication was subject to stock-outs, as procurement protocols had not accounted for supplies needed to de-worm the adolescent population in the HPV-Plus program. Constraint of health care workers, especially when attempting to serve large schools in one day, was noted. Feasibility and acceptance reflect one region only.

^1^Bold text indicates heading of factor as presented in the Theory of Change.

### 3.7 Rwanda

Rwanda was the first low-income country to introduce HPV vaccine at the national level, using a phased approach. The one included article reports outcomes from the initial, 2011 phase of introduction, also describing context and mechanisms used in national scale-up through 2014 (Table 1) [33], which continue to influence the current program. Context included a small proportion of out-of-school girls, who were directed to present to local health facilities for vaccination. The article does not suggest these girls received other integrated services. The involvement and support of Rwanda's then-First Lady provides an example of government leader endorsement. Delivery context involved school-based, campaign-style delivery that worked through a mechanism of creating novel “health days”, embedded into the primary health care system, where teachers and local health care professionals collaborated to educate girls being vaccinated on topics such as hygiene, nutrition, infectious diseases, and reproductive health [33]. The article also clearly describes the mechanism of multi-sectoral cooperation for policy and planning, at Ministry and National Immunization Technical Advisory Group (NITAG) levels, which created new technical working groups of experts in education, gender and family promotion, and cancer care. Multi-sectoral collaboration extended to programme planning and readiness, cold chain logistics, vaccine procurement, target population enumeration, and data collection. Reported outcomes included high rates of coverage. However, study design did not support attribution of effect.

### 3.8 South Africa

An integrated approach from one sub-national district in 2011 in South Africa (Table 1) used school-based outreach delivery in the socio-cultural context of a high HIV/AIDS prevalence and government endorsement, benefiting from the context of a national policy and practice guideline that had recently been put into place supporting an integrated approach [34]. The integrated approach was embedded in a school-based primary health care package of services incorporating novel and existing components [34], through simultaneous delivery with other services, including a general clinical and nutritional appraisal, by existing school health staff. We categorized the emphasis on shared staffing as a cost-sharing mechanism. This somewhat limited case reported the outcome of an increase in number of vaccinated girls. This article provides a number of negative or “counter-factual” examples, with authors reporting lack of cold chain integration in the setting of challenges with storage capacity; lack of integrated enumeration in the setting of challenges with correct identification of girls for follow-up doses; and lack of integration of data capture into existing school health programs in the setting of programme monitoring challenges due to the use of separate, paper-based vaccine records. This set of “counter-factual” examples supported a revision of our TOC that includes these mechanisms.

### 3.9 Tanzania

Tanzania integrated HPV vaccine in schools within a novel “HPV-Plus” package of adolescent primary health care screening, deworming, and educational content (Table 2) delivered across six sub-national districts starting in 2020 [26] (Table 1). Facility-based delivery of the package did not meet feasibility criteria; services were delivered in a school-based outreach delivery context. The programme addressed the context of communities affected by HIV/AIDS in planning efforts; addressed the context of potential gender inequity by offering an educational package for boys as well as girls; and benefited from the context of a national policy that had been put into place supporting an integrated approach [26]. The country used the mechanism of multi-sectoral cooperation, wherein planning and monitoring activities were integrated between the vaccine programme and ongoing broader primary health care efforts. Preferences of adolescents and parents were explicitly incorporated into programme design (Fig. 2), through a participatory human-centered design methodology. The programme was embedded into the routine immunization program; of note, this integrated approach has been scaled up to additional regions in the current program. Existing routine immunization workers simultaneously delivered HPV vaccines with the integrated service package, which we categorized as a cost-sharing mechanism. The program also leveraged supply chain and distribution systems as well as communications from both school health and primary health care / routine immunization programmes. The article reported increases in HPV vaccine coverage, and, uniquely among other articles, reported increased participation with the integrated services, particularly nutrition and vision screening. Additional outcomes included positive attitudes toward the integrated services, and costs, although a detailed cost comparison was not performed. The article did report challenges with stock-out of the deworming medication included in the package, noting inadequate integration of procurement mechanisms. Various staffing constraints were also noted. TOC iterations captured these challenges as “comparative programme efficiencies”.

### 3.10 Togo

Togo conducted a sub-national two-district pilot in 2016 and 2017 that integrated a novel age-appropriate adolescent health educational curriculum on puberty education, menstrual hygiene and hand-washing with the simultaneous delivery of HPV vaccination in a school-based campaign approach [11]. The programme addressed the context of potential gender inequity by providing the educational package to boys, and responded to the context of needing to serve out-of-school girls with a tailored outreach strategy. Responsiveness to these factors seems to have been enabled by the use of a multi-sectoral cooperation mechanism for planning. The intervention was embedded in the school health program, and delivered by a differing configuration of workers (teachers and vaccinators in one, vaccinators only in the other). The article reported a favorable effect on HPV vaccine coverage, but study design did not support attribution of effect to an integrated approach. The paper did report positive attitudes from multiple stakeholders in response to the integration experience, and presented evidence of a positive impact of the integrated approach on youth participation in the educational sessions, and, uniquely, on youth knowledge and recall of session content.

### 3.11 Uganda

Uganda conducted a sub-national pilot in 2009, using schools as delivery sites for HPV vaccine alongside deworming medicines and vitamin A in one district through an existing community-based “Child Days Plus” programme [31,32]. The programme responded to the context of needing to serve out-of-school girls by referring to facilities or other routine mop-up services; neither paper suggests these girls received the other integrated services. Local government did endorse and promote the integrated approach, which one article linked to increased buy-in among health care and education workers [32]. The programme was embedded into routine immunization services, sharing costs by using immunizers to deliver the integrated package, and leveraging existing immunization progamme cold chain, supply and distribution routes for HPV vaccine shipments [32]. Both articles documented relatively high initial coverage over approximately one year, but neither article was designed to accurately assess a coverage effect due to an integrated approach – differences could not be parsed from the use of an age-based versus a grade-based approach to enumerate the target population. One article did report experiences with programme dropout and school disruptions [32]; TOC iterations captured these as an “efficiency” concept.

## 4 Discussion

### 4.1 Summary of main findings

To our knowledge this is the first realist synthesis describing integrated approaches to delivery of HPV vaccines and other adolescent health services. Our iterative realist process yielded an iterated Theory of Change (TOC) developed with guidance from policy, programme and research experts in the field. A truly linear and connected TOC model is not yet possible, given the diversity of potential interventions and the limited set of evidence available. Instead, our TOC is necessarily more open and allows mapping of potentially beneficial combinations of context and mechanisms, as well as where there are major gaps in the current evidence base. This theory can guide researcher and practitioner efforts to fill current evidence gaps in understanding how and why integrated approaches may yield reported results in this space. By examining available country experience, we have also described how varying combinations of context, mechanisms and outcomes have been implemented and evaluated in real-world settings.

Our synthesis, captured in TOC iterations, has notable implications for future research and practice. While integrated approaches were largely delivered to a population of in-school 9–10 year olds, the more recently published experiences addressed wider ranges, up to 13 or 14 years (Togo and Tanzania). As many programs shift to a 9 year old target age, future integration efforts will need to distinguish health needs of younger compared to older adolescents. While most countries described some level of strategy to reach out of school girls, data on reach and effectiveness of integrated mechanisms to meet their needs was sparse. Future research and practice should address this significant gap, as out-of-school girls are likely to have unique health needs [35]. Despite growing consensus that HPV vaccine programs should emphasize strategies for adolescents with HIV [36], we found little evidence of programme response to this context. We did see evidence of countries acknowledging the context of gender inequity, generally through explicit inclusion of boys in programming; those papers also disaggregated relevant outcomes by gender. When the local context involved government endorsement, it took two forms: that of a new, national policy, where HPV vaccine was integrated with a novel package of services; or of public messages of governmental support for an integrated approach, in which case HPV vaccine was added to an existing package of services.

Integrated packages included a limited range of other services, ranging from booster doses of other childhood vaccines (standard national schedule boosters of Measles, Rubella and Diptheria Tetanus administered to seven year olds as part of the school health services program) in Malaysia; deworming medications in Tanzania and Uganda; nutrition and development screening in Malaysia, Tanzania, and South Africa; vitamin A in Uganda; and primary care or sexual/reproductive health programming in Malaysia, Rwanda, Togo, and to some extent in Tanzania. All of these options had been described in a 2013 comprehensive review of evidence-based options [9]; thus, this guidance remains relevant, and countries should develop integrated service packages according to the context of local needs, available existing programs to leverage, and emerging national priorities. While each country used multiple integrated mechanisms, the use of a multisectoral cooperation mechanism appeared to be linked to the use of a large number of other mechanisms, including the integration of cost-sharing, supply chain and distribution, and programme monitoring, as seen particularly in Malaysia, Tanzania and Togo. We note recent attention to this concept in other HPV vaccine literature [37].

Our findings highlight the importance of studying not just coverage, but outcomes related to understanding of and confidence in public health interventions, such as knowledge and acceptability of an integrated approach. Understanding the effect of an integrated service delivery package on such outcomes is critical to understanding how integrated approaches may work, to vaccines and across a broader context [7,38,39].

### 4.2 Policy and Practice implications for the implementation research agenda

To our knowledge, three of the included countries have continued, or scaled up, their integrated approaches – Malaysia, Tanzania and Rwanda, each integrating into a primary health care package of services for adolescents, using school-based delivery. We show a clear need to design integrated interventions to measure outcomes in a way that allows attribution of effect (e.g. if change in uptake is truly due to an integrated mechanism, instead of enhanced demand generation). We note particular gaps in the evidence around outcomes of integration for priority populations with unique needs, such as adolescents living with HIV, out of school, or otherwise not consistently reached by routine health services (e.g. in migratory or conflict-affected communities). Gains in school attendance over recent decades are in decline, particularly among adolescents [40,41], and many out of school youth are increasingly concentrated in small subnational communities [42]. Thus, there is also a clear agenda for studying integrated approaches through subnational studies. We also note a gap in knowledge on how integrated packages may be delivered through community and health facility access points. Closing these gaps will require mixed methods, perhaps drawing on realist evaluation concepts, to understand underlying drivers of success or failure of an integrated approach.

Finally, an integrated approach is often cited as a means to improve efficiency and reach, and contribute to programme sustainability [39,43,44]. However, we found no usable data on costs – a core component of sustainability. We recommend that the research agenda include health economics perspectives and objectives, so that costs, and potential variance with an integrated approach, are measured appropriately and comprehensively.

### 4.3 Strengths and limitations

An important strength of this review is its flexible, pragmatic realist synthesis methodology, aimed not at an exhaustive review of the literature, but on providing a practical framework ready for use by practitioners. We note increasing use of this methodology to synthesize evidence on complex interventions [[45], [46], [47]] – to help describe in practical terms not only whether an intervention works, but how, and under what conditions. This methodology permitted us to admit evidence of lesser quality, including project evaluations and grey literature, harnessing learnings that may otherwise be overlooked. Consistent with other realist synthesis experiences, we noted that context and mechanism factors were often intertwined and resistant to separation [48], making it difficult to parse context and mechanism factor effects on particular outcomes. Therefore, we cannot recommend specific combinations of context and mechanism to yield an outcome of interest. Rather, our TOC provides a framework to guide the incorporation of these multiple factors into programme, policy, and research protocol design. Our findings are drawn from a notably small number of countries (six) representing a limited geography (five in Africa, one in Southeast Asia), limiting the transferability of challenges and opportunities we report. An additional limitation involves our dependence upon article availability. Not all country experience with integrated approaches has been published, even in the grey literature that we with our commissioner experts considered. Many articles screened lacked sufficient data to extract to inform our TOC [7,[49], [50], [51]]. And, perhaps because of the prior absence of a TOC framework, many included articles did not focus on evaluating how and why an integrated approach might work.

## 5 Conclusions and recommendations

This realist synthesis review helps to describe and fill important gaps in available evidence on how integrated approaches might effectively yield outcomes of interest for HPV vaccines and other adolescent health services. We call on implementation researchers and funders to invest in more rigorous study designs that can provide more definitive insights into how integrated approaches in varying contexts may be linked to vaccination outcomes – particularly coverage. Our synthesis highlights the need to evaluate how an integrated approach may address programmatic gender inequities, and meet unique needs of locally relevant underserved populations including out of school youth, those affected by HIV, migration, or conflict, and others. Our iterated TOC can guide future implementation research, particularly on the effect an integrated approach may have on other adolescent health services, health knowledge and acceptability. While our findings are oriented to HPV vaccination programs, they have relevance to a broader package of primary health care services, including other vaccines for adolescents. The grounding of our proposed TOC in country experiences will help programme implementers, policy-makers, implementation scientists, and funders base new efforts in a practical theoretical framework – and prioritize filling key gaps in the existing evidence base.

## Funding

CM and MCJ contributions to this work were supported by the Bill & Melinda Gates Foundation [grant number INV-006006].

CW and SN contributions to this work were made possible by the generous support of the American people through the U.S. Agency for International Development (USAID) under the terms of the Cooperative Agreement [#7200AA20CA00002], led by Jhpiego and partners. The contents of this manuscript represent the views and opinions of the authors and do not necessarily reflect the views and opinions of the U.S. Agency for International Development (USAID) or the United States Government.

Although one commissioner is employed by Public Health Institute, via USAID's Global Health Training, Advisory and Support Contract (GHTASC), that role was on an individual basis. The Sponsors were not involved in study design, data collection, analysis or interpretation of data, or in the writing of this manuscript report and decision to submit for publication.

## CRediT authorship contribution statement

**Mary Carol Jennings:** Writing – review & editing, Writing – original draft, Project administration, Methodology, Investigation, Formal analysis, Data curation, Conceptualization. **Sarah Nabia:** Writing – review & editing, Writing – original draft, Visualization, Investigation, Formal analysis, Data curation. **Christopher Morgan:** Writing – review & editing, Supervision, Methodology, Funding acquisition, Conceptualization. **Chinelo Cynthia Nduka:** Writing – original draft, Formal analysis, Data curation. **Julia Brotherton:** Writing – review & editing, Validation. **Megan Holloway:** Writing – review & editing, Validation. **Katharine Bagshaw:** Writing – review & editing, Validation. **Paul Bloem:** Writing – review & editing, Validation. **Chizoba Wonodi:** Writing – review & editing, Writing – original draft, Supervision, Methodology, Funding acquisition, Conceptualization.

## Declaration of competing interest

The authors declare the following financial interests/personal relationships which may be considered as potential competing interests: Two authors are also co-authors on two of the articles retained in the final analysis [8, 25] – P.B., C.M. The remaining authors declare that they have no known competing financial interests or personal relationships that could have appeared to influence the work reported in this paper.

## Data Availability

Data will be made available on request.

## Appendix A Supplementary data

Supplementary material 1 Image 1

Supplementary material 2 Image 2
