# Supplementary material for: Realist review of low- to upper-middle-income country experiences on integration of HPV vaccination with other adolescent health services
Source: Vaccine. 2025 Mar 19;50:None. doi: 10.1016/j.vaccine.2025.126833 (PMC11878278; doi:10.1016/j.vaccine.2025.126833)
Supplement: Supplementary file 1 — Supplementary material 1 [file mmc1.docx]

## **Appendix I: Peer Reviewed Database Search Strategy**

PubMed

(("Papillomavirus Vaccines"[Mesh] OR ((HPV[tiab] OR "human papillomavirus"[tiab]) AND "immunization programs"[mesh]) OR "HPV vaccin*"[tiab] OR "human papillomavirus vaccin*"[tiab] OR "HPV immuniz*"[tiab] OR "human papillomavirus immuniz*"[tiab] OR "HPV immunis*"[tiab] OR "human papillomavirus immunis*"[tiab])

AND

(("Adolescent Health Services"[Mesh]) OR ((("Adolescent"[Mesh]) OR "Child"[Mesh:NoExp] OR adolescen*[tiab] OR youth*[tiab] OR "young adult*"[tiab] OR "young person*"[tiab] OR "young women"[tiab] OR "young woman"[tiab] OR teen*[tiab] OR girl*[tiab] OR "school age"[tiab]) AND ("Health Services"[Mesh] OR "Health Education"[Mesh] OR "health service*"[tiab] OR "healthcare"[tiab] OR "health care"[tiab] OR "health education"[tiab] OR "preventive health"[tiab] OR "promotive health"[tiab]))))

AND

("afghanistan"[MeSH Terms] OR "albania"[MeSH Terms] OR "algeria"[MeSH Terms] OR "american samoa"[MeSH Terms] OR "angola"[MeSH Terms] OR "antigua and barbuda"[MeSH Terms] OR "argentina"[MeSH Terms] OR "armenia"[MeSH Terms] OR "aruba"[MeSH Terms] OR "azerbaijan"[MeSH Terms] OR "bahrain"[MeSH Terms] OR "bangladesh"[MeSH Terms] OR "barbados"[MeSH Terms] OR "republic of belarus"[MeSH Terms] OR "belize"[MeSH Terms] OR "benin"[MeSH Terms] OR "bhutan"[MeSH Terms] OR "bolivia"[MeSH Terms] OR "bosnia and herzegovina"[MeSH Terms] OR "botswana"[MeSH Terms] OR "brazil"[MeSH Terms] OR "bulgaria"[MeSH Terms] OR "burkina faso"[MeSH Terms] OR "burundi"[MeSH Terms] OR "cabo verde"[MeSH Terms] OR "cambodia"[MeSH Terms] OR "cameroon"[MeSH Terms] OR "central african republic"[MeSH Terms] OR "chad"[MeSH Terms] OR "chile"[MeSH Terms] OR "china"[MeSH Terms] OR "colombia"[MeSH Terms] OR "comoros"[MeSH Terms] OR "democratic republic of the congo"[MeSH Terms] OR "congo"[MeSH Terms] OR "costa rica"[MeSH Terms] OR "cote d ivoire"[MeSH Terms] OR "croatia"[MeSH Terms] OR "cuba"[MeSH Terms] OR "cyprus"[MeSH Terms] OR "czech republic"[MeSH Terms] OR "djibouti"[MeSH Terms] OR "dominica"[MeSH Terms] OR "dominican republic"[MeSH Terms] OR "ecuador"[MeSH Terms] OR "egypt"[MeSH Terms] OR "el salvador"[MeSH Terms] OR "equatorial guinea"[MeSH Terms] OR "eritrea"[MeSH Terms] OR "estonia"[MeSH Terms] OR "eswatini"[MeSH Terms] OR "eswatini"[MeSH Terms] OR "ethiopia"[MeSH Terms] OR "fiji"[MeSH Terms] OR "gabon"[MeSH Terms] OR "gambia"[MeSH Terms] OR "georgia republic"[MeSH Terms] OR "ghana"[MeSH Terms] OR "gibraltar"[MeSH Terms] OR "grenada"[MeSH Terms] OR "guam"[MeSH Terms] OR "guatemala"[MeSH Terms] OR "guinea"[MeSH Terms] OR "guinea bissau"[MeSH Terms] OR "guyana"[MeSH Terms] OR "haiti"[MeSH Terms] OR "honduras"[MeSH Terms] OR "india"[MeSH Terms] OR "indonesia"[MeSH Terms] OR "iran"[MeSH Terms] OR "iraq"[MeSH Terms] OR "jamaica"[MeSH Terms] OR "jordan"[MeSH Terms] OR "kazakhstan"[MeSH Terms] OR "kenya"[MeSH Terms] OR "democratic people's republic of korea"[MeSH Terms] OR "republic of korea"[MeSH Terms] OR "kosovo"[MeSH Terms] OR "kyrgyzstan"[MeSH Terms] OR "laos"[MeSH Terms] OR "latvia"[MeSH Terms] OR "lebanon"[MeSH Terms] OR "lesotho"[MeSH Terms] OR "liberia"[MeSH Terms] OR "libya"[MeSH Terms] OR "lithuania"[MeSH Terms] OR "macau"[MeSH Terms] OR "republic of north macedonia"[MeSH Terms] OR "madagascar"[MeSH Terms] OR "malawi"[MeSH Terms] OR "malaysia"[MeSH Terms] OR "indian ocean islands"[MeSH Terms] OR "mali"[MeSH Terms] OR "malta"[MeSH Terms] OR "micronesia"[MeSH Terms] OR "palau"[MeSH Terms] OR "mauritania"[MeSH Terms] OR "mauritius"[MeSH Terms] OR "mexico"[MeSH Terms] OR "moldova"[MeSH Terms] OR "mongolia"[MeSH Terms] OR "montenegro"[MeSH Terms] OR "morocco"[MeSH Terms] OR "mozambique"[MeSH Terms] OR "myanmar"[MeSH Terms] OR "namibia"[MeSH Terms] OR "nepal"[MeSH Terms] OR "netherlands antilles"[MeSH Terms] OR "nicaragua"[MeSH Terms] OR "niger"[MeSH Terms] OR "nigeria"[MeSH Terms] OR "oman"[MeSH Terms] OR "pakistan"[MeSH Terms] OR "panama"[MeSH Terms] OR "papua new guinea"[MeSH Terms] OR "paraguay"[MeSH Terms] OR "peru"[MeSH Terms] OR "philippines"[MeSH Terms] OR "poland"[MeSH Terms] OR "portugal"[MeSH Terms] OR "puerto rico"[MeSH Terms] OR "romania"[MeSH Terms] OR "russia"[MeSH Terms] OR "rwanda"[MeSH Terms] OR "samoa"[MeSH Terms] OR "sao tome and principe"[MeSH Terms] OR "saudi arabia"[MeSH Terms] OR "senegal"[MeSH Terms] OR "serbia"[MeSH Terms] OR "seychelles"[MeSH Terms] OR "sierra leone"[MeSH Terms] OR "slovakia"[MeSH Terms] OR "slovenia"[MeSH Terms] OR "melanesia"[MeSH Terms] OR "somalia"[MeSH Terms] OR "south africa"[MeSH Terms] OR "south sudan"[MeSH Terms] OR "sri lanka"[MeSH Terms] OR "saint kitts and nevis"[MeSH Terms] OR "saint lucia"[MeSH Terms] OR "saint vincent and the grenadines"[MeSH Terms] OR "sudan"[MeSH Terms] OR "suriname"[MeSH Terms] OR "syria"[MeSH Terms] OR "tajikistan"[MeSH Terms] OR "tanzania"[MeSH Terms] OR "thailand"[MeSH Terms] OR "timor leste"[MeSH Terms] OR "togo"[MeSH Terms] OR "tonga"[MeSH Terms] OR "trinidad and tobago"[MeSH Terms] OR "tunisia"[MeSH Terms] OR "turkey"[MeSH Terms] OR "turkmenistan"[MeSH Terms] OR "uganda"[MeSH Terms] OR "ukraine"[MeSH Terms] OR "uruguay"[MeSH Terms] OR "uzbekistan"[MeSH Terms] OR "vanuatu"[MeSH Terms] OR "venezuela"[MeSH Terms] OR "vietnam"[MeSH Terms] OR "middle east"[MeSH Terms] OR "yemen"[MeSH Terms] OR "yugoslavia"[MeSH Terms] OR "zambia"[MeSH Terms] OR "zimbabwe"[MeSH Terms] OR "africa south of the sahara"[MeSH Terms] OR "africa, central"[MeSH Terms] OR "africa, northern"[MeSH Terms] OR "africa, southern"[MeSH Terms] OR "africa, eastern"[MeSH Terms] OR "africa, western"[MeSH Terms] OR "west indies"[MeSH Terms] OR "indian ocean islands"[MeSH Terms] OR "caribbean region"[MeSH Terms] OR "central america"[MeSH Terms] OR "latin america"[MeSH Terms] OR "south america"[MeSH Terms] OR "asia, central"[MeSH Terms] OR "asia, northern"[MeSH Terms] OR "asia, southeastern"[MeSH Terms] OR "asia, western"[MeSH Terms] OR "europe, eastern"[MeSH Terms] OR "developing countries"[MeSH Terms] OR "afghanistan"[Text Word] OR "albania"[Text Word] OR "algeria"[Text Word] OR "american samoa"[Text Word] OR "angola"[Text Word] OR "antigua"[Text Word] OR "barbuda"[Text Word] OR "argentina"[Text Word] OR "armenia"[Text Word] OR "armenian"[Text Word] OR "aruba"[Text Word] OR "azerbaijan"[Text Word] OR "bahrain"[Text Word] OR "bangladesh"[Text Word] OR "barbados"[Text Word] OR "belarus"[Text Word] OR "byelarus"[Text Word] OR "belorussia"[Text Word] OR "byelorussian"[Text Word] OR "belize"[Text Word] OR "british honduras"[Text Word] OR "benin"[Text Word] OR "dahomey"[Text Word] OR "bhutan"[Text Word] OR "bolivia"[Text Word] OR "bosnia"[Text Word] OR "herzegovina"[Text Word] OR "botswana"[Text Word] OR "bechuanaland"[Text Word] OR "brazil"[Text Word] OR "brasil"[Text Word] OR "bulgaria"[Text Word] OR "burkina faso"[Text Word] OR "burkina fasso"[Text Word] OR "upper volta"[Text Word] OR "burundi"[Text Word] OR "urundi"[Text Word] OR "cabo verde"[Text Word] OR "cape verde"[Text Word] OR "cambodia"[Text Word] OR "kampuchea"[Text Word] OR "khmer republic"[Text Word] OR "cameroon"[Text Word] OR "cameron"[Text Word] OR "cameroun"[Text Word] OR "central african republic"[Text Word] OR "ubangi shari"[Text Word] OR "chad"[Text Word] OR "chile"[Text Word] OR "china"[Text Word] OR "colombia"[Text Word] OR "comoros"[Text Word] OR "comoro islands"[Text Word] OR "mayotte"[Text Word] OR "congo"[Text Word] OR "zaire"[Text Word] OR "costa rica"[Text Word] OR "cote d ivoire"[Text Word] OR "cote d ivoire"[Text Word] OR "cote d ivoire"[Text Word] OR "ivory coast"[Text Word] OR "croatia"[Text Word] OR "cuba"[Text Word] OR "cyprus"[Text Word] OR "czech republic"[Text Word] OR "czechoslovakia"[Text Word] OR "djibouti"[Text Word] OR "french somaliland"[Text Word] OR "dominica"[Text Word] OR "dominican republic"[Text Word] OR "ecuador"[Text Word] OR "egypt"[Text Word] OR "united arab republic"[Text Word] OR "el salvador"[Text Word] OR "equatorial guinea"[Text Word] OR "spanish guinea"[Text Word] OR "eritrea"[Text Word] OR "estonia"[Text Word] OR "eswatini"[Text Word] OR "swaziland"[Text Word] OR "ethiopia"[Text Word] OR "fiji"[Text Word] OR "gabon"[Text Word] OR "gabonese republic"[Text Word] OR "gambia"[Text Word] OR "georgia"[Text Word] OR "georgian"[Text Word] OR "ghana"[Text Word] OR "gold coast"[Text Word] OR "gibraltar"[Text Word] OR "grenada"[Text Word] OR "guam"[Text Word] OR "guatemala"[Text Word] OR "guinea"[Text Word] OR "guyana"[Text Word] OR "guiana"[Text Word] OR "haiti"[Text Word] OR "hispaniola"[Text Word] OR "honduras"[Text Word] OR "india"[Text Word] OR "indonesia"[Text Word] OR "timor"[Text Word] OR "iran"[Text Word] OR "iraq"[Text Word] OR "isle of man"[Text Word] OR "jamaica"[Text Word] OR "jordan"[Text Word] OR "kazakhstan"[Text Word] OR "kazakh"[Text Word] OR "kenya"[Text Word] OR "korea"[Text Word] OR "kosovo"[Text Word] OR "kyrgyzstan"[Text Word] OR "kirghizia"[Text Word] OR "kirgizstan"[Text Word] OR "kyrgyz republic"[Text Word] OR "kirghiz"[Text Word] OR "laos"[Text Word] OR "lao pdr"[Text Word] OR "lao people s democratic republic"[Text Word] OR "latvia"[Text Word] OR "lebanon"[Text Word] OR "lesotho"[Text Word] OR "basutoland"[Text Word] OR "liberia"[Text Word] OR "libya"[Text Word] OR "libyan arab jamahiriya"[Text Word] OR "lithuania"[Text Word] OR "macau"[Text Word] OR "macao"[Text Word] OR "macedonia"[Text Word] OR "madagascar"[Text Word] OR "malagasy republic"[Text Word] OR "malawi"[Text Word] OR "nyasaland"[Text Word] OR "malaysia"[Text Word] OR "maldives"[Text Word] OR "indian ocean"[Text Word] OR "mali"[Text Word] OR "malta"[Text Word] OR "micronesia"[Text Word] OR "kiribati"[Text Word] OR "marshall islands"[Text Word] OR "nauru"[Text Word] OR "northern mariana islands"[Text Word] OR "palau"[Text Word] OR "tuvalu"[Text Word] OR "mauritania"[Text Word] OR "mauritius"[Text Word] OR "mexico"[Text Word] OR "moldova"[Text Word] OR "moldovian"[Text Word] OR "mongolia"[Text Word] OR "montenegro"[Text Word] OR "morocco"[Text Word] OR "ifni"[Text Word] OR "mozambique"[Text Word] OR "portuguese east africa"[Text Word] OR "myanmar"[Text Word] OR "burma"[Text Word] OR "namibia"[Text Word] OR "nepal"[Text Word] OR "netherlands antilles"[Text Word] OR "nicaragua"[Text Word] OR "niger"[Text Word] OR "nigeria"[Text Word] OR "oman"[Text Word] OR "muscat"[Text Word] OR "pakistan"[Text Word] OR "panama"[Text Word] OR "papua new guinea"[Text Word] OR "paraguay"[Text Word] OR "peru"[Text Word] OR "philippines"[Text Word] OR "philipines"[Text Word] OR "phillipines"[Text Word] OR "phillippines"[Text Word] OR "poland"[Text Word] OR "polish people s republic"[Text Word] OR "portugal"[Text Word] OR "portuguese republic"[Text Word] OR "puerto rico"[Text Word] OR "romania"[Text Word] OR "russia"[Text Word] OR "russian federation"[Text Word] OR "ussr"[Text Word] OR "soviet union"[Text Word] OR "union of soviet socialist republics"[Text Word] OR "rwanda"[Text Word] OR "ruanda"[Text Word] OR "samoa"[Text Word] OR "pacific islands"[Text Word] OR "polynesia"[Text Word] OR "samoan islands"[Text Word] OR "sao tome and principe"[Text Word] OR "saudi arabia"[Text Word] OR "senegal"[Text Word] OR "serbia"[Text Word] OR "seychelles"[Text Word] OR "sierra leone"[Text Word] OR "slovakia"[Text Word] OR "slovak republic"[Text Word] OR "slovenia"[Text Word] OR "melanesia"[Text Word] OR "solomon island"[Text Word] OR "solomon islands"[Text Word] OR "norfolk island"[Text Word] OR "somalia"[Text Word] OR "south africa"[Text Word] OR "south sudan"[Text Word] OR "sri lanka"[Text Word] OR "ceylon"[Text Word] OR "saint kitts and nevis"[Text Word] OR "st kitts and nevis"[Text Word] OR "saint lucia"[Text Word] OR "st lucia"[Text Word] OR "saint vincent"[Text Word] OR "st vincent"[Text Word] OR "grenadines"[Text Word] OR "sudan"[Text Word] OR "suriname"[Text Word] OR "surinam"[Text Word] OR "syria"[Text Word] OR "syrian arab republic"[Text Word] OR "tajikistan"[Text Word] OR "tadjikistan"[Text Word] OR "tadzhikistan"[Text Word] OR "tadzhik"[Text Word] OR "tanzania"[Text Word] OR "tanganyika"[Text Word] OR "thailand"[Text Word] OR "siam"[Text Word] OR "timor leste"[Text Word] OR "east timor"[Text Word] OR "togo"[Text Word] OR "togolese republic"[Text Word] OR "tonga"[Text Word] OR "trinidad"[Text Word] OR "tobago"[Text Word] OR "tunisia"[Text Word] OR "turkey"[Text Word] OR "turkmenistan"[Text Word] OR "turkmen"[Text Word] OR "uganda"[Text Word] OR "ukraine"[Text Word] OR "uruguay"[Text Word] OR "uzbekistan"[Text Word] OR "uzbek"[Text Word] OR "vanuatu"[Text Word] OR "new hebrides"[Text Word] OR "venezuela"[Text Word] OR "vietnam"[Text Word] OR "viet nam"[Text Word] OR "middle east"[Text Word] OR "west bank"[Text Word] OR "gaza"[Text Word] OR "palestine"[Text Word] OR "yemen"[Text Word] OR "yugoslavia"[Text Word] OR "zambia"[Text Word] OR "zimbabwe"[Text Word] OR "northern rhodesia"[Text Word] OR "global south"[Text Word] OR "africa south of the sahara"[Text Word] OR "sub saharan africa"[Text Word] OR "subsaharan africa"[Text Word] OR "central africa"[Text Word] OR "north africa"[Text Word] OR "northern africa"[Text Word] OR "magreb"[Text Word] OR "maghrib"[Text Word] OR "sahara"[Text Word] OR "southern africa"[Text Word] OR "east africa"[Text Word] OR "eastern africa"[Text Word] OR "west africa"[Text Word] OR "western africa"[Text Word] OR "west indies"[Text Word] OR "indian ocean islands"[Text Word] OR "caribbean"[Text Word] OR "central america"[Text Word] OR "latin america"[Text Word] OR "south america"[Text Word] OR "central asia"[Text Word] OR "north asia"[Text Word] OR "northern asia"[Text Word] OR "southeastern asia"[Text Word] OR "south eastern asia"[Text Word] OR "southeast asia"[Text Word] OR "south east asia"[Text Word] OR "western asia"[Text Word] OR "east europe"[Text Word] OR "eastern europe"[Text Word] OR "developing country"[Text Word] OR "developing countries"[Text Word] OR "developing nation"[Text Word] OR "developing nations"[Text Word] OR "developing population"[Text Word] OR "developing populations"[Text Word] OR "developing world"[Text Word] OR "less developed country"[Text Word] OR "less developed countries"[Text Word] OR "less developed nation"[Text Word] OR "less developed nations"[Text Word] OR "less developed world"[Text Word] OR "lesser developed countries"[Text Word] OR "lesser developed nations"[Text Word] OR "under developed country"[Text Word] OR "under developed countries"[Text Word] OR "under developed nations"[Text Word] OR "under developed world"[Text Word] OR "underdeveloped country"[Text Word] OR "underdeveloped countries"[Text Word] OR "underdeveloped nation"[Text Word] OR "underdeveloped nations"[Text Word] OR "underdeveloped population"[Text Word] OR "underdeveloped populations"[Text Word] OR "underdeveloped world"[Text Word] OR "middle income country"[Text Word] OR "middle income countries"[Text Word] OR "middle income nation"[Text Word] OR "middle income nations"[Text Word] OR "middle income population"[Text Word] OR "middle income populations"[Text Word] OR "low income country"[Text Word] OR "low income countries"[Text Word] OR "low income nation"[Text Word] OR "low income nations"[Text Word] OR "low income population"[Text Word] OR "low income populations"[Text Word] OR "lower income country"[Text Word] OR "lower income countries"[Text Word] OR "lower income nations"[Text Word] OR "lower income population"[Text Word] OR "lower income populations"[Text Word] OR "underserved countries"[Text Word] OR "underserved nations"[Text Word] OR "underserved population"[Text Word] OR "underserved populations"[Text Word] OR "under served population"[Text Word] OR "under served populations"[Text Word] OR "deprived countries"[Text Word] OR "deprived population"[Text Word] OR "deprived populations"[Text Word] OR "poor country"[Text Word] OR "poor countries"[Text Word] OR "poor nation"[Text Word] OR "poor nations"[Text Word] OR "poor population"[Text Word] OR "poor populations"[Text Word] OR "poor world"[Text Word] OR "poorer countries"[Text Word] OR "poorer nations"[Text Word] OR "poorer population"[Text Word] OR "poorer populations"[Text Word] OR "developing economy"[Text Word] OR "developing economies"[Text Word] OR "less developed economy"[Text Word] OR "less developed economies"[Text Word] OR "underdeveloped economies"[Text Word] OR "middle income economy"[Text Word] OR "middle income economies"[Text Word] OR "low income economy"[Text Word] OR "low income economies"[Text Word] OR "lower income economies"[Text Word] OR "low gdp"[Text Word] OR "low gnp"[Text Word] OR "low gross domestic"[Text Word] OR "low gross national"[Text Word] OR "lower gdp"[Text Word] OR "lower gross domestic"[Text Word] OR "lmic"[Text Word] OR "lmics"[Text Word] OR "third world"[Text Word] OR "lami country"[Text Word] OR "lami countries"[Text Word] OR "transitional country"[Text Word] OR "transitional countries"[Text Word] OR "emerging economies"[Text Word] OR "emerging nation"[Text Word] OR "emerging nations"[Text Word])

Embase

'wart virus vaccine'/exp OR ((hpv:ti,ab OR 'human papillomavirus':ti,ab) AND 'preventive health service'/exp) OR 'hpv vaccin*':ti,ab OR 'human papillomavirus vaccin*':ti,ab OR 'hpv immuniz*':ti,ab OR 'human papillomavirus immuniz*':ti,ab OR 'hpv immunis*':ti,ab OR 'human papillomavirus immunis*':ti,ab

AND

'child health care'/exp OR (('adolescent'/exp OR 'child'/de OR adolescen*:ti,ab OR youth*:ti,ab OR 'young adult*':ti,ab OR 'young person*':ti,ab OR 'young women':ti,ab OR 'young woman':ti,ab OR teen*:ti,ab OR girl*:ti,ab OR 'school age':ti,ab) AND ('health service'/exp OR 'health education'/exp OR 'health service*':ti,ab OR 'healthcare':ti,ab OR 'health care':ti,ab OR 'health education':ti,ab OR 'preventive health':ti,ab OR 'promotive health':ti,ab))

AND

'developing country':ab,ti OR 'developing countries':ab,ti OR 'developing nation':ab,ti OR 'developing nations':ab,ti OR 'developing population':ab,ti OR 'developing populations':ab,ti OR 'developing world':ab,ti OR 'less developed country':ab,ti OR 'less developed countries':ab,ti OR 'less developed nation':ab,ti OR 'less developed nations':ab,ti OR 'less developed population':ab,ti OR 'less developed populations':ab,ti OR 'less developed world':ab,ti OR 'lesser developed country':ab,ti OR 'lesser developed countries':ab,ti OR 'lesser developed nation':ab,ti OR 'lesser developed nations':ab,ti OR 'lesser developed population':ab,ti OR 'lesser developed populations':ab,ti OR 'lesser developed world':ab,ti OR 'under developed country':ab,ti OR 'under developed countries':ab,ti OR 'under developed nation':ab,ti OR 'under developed nations':ab,ti OR 'under developed population':ab,ti OR 'under developed populations':ab,ti OR 'under developed world':ab,ti OR 'underdeveloped country':ab,ti OR 'underdeveloped countries':ab,ti OR 'underdeveloped nation':ab,ti OR 'underdeveloped nations':ab,ti OR 'underdeveloped population':ab,ti OR 'underdeveloped populations':ab,ti OR 'underdeveloped world':ab,ti OR 'middle income country':ab,ti OR 'middle income countries':ab,ti OR 'middle income nation':ab,ti OR 'middle income nations':ab,ti OR 'middle income population':ab,ti OR 'middle income populations':ab,ti OR 'low income country':ab,ti OR 'low income countries':ab,ti OR 'low income nation':ab,ti OR 'low income nations':ab,ti OR 'low income population':ab,ti OR 'low income populations':ab,ti OR 'lower income country':ab,ti OR 'lower income countries':ab,ti OR 'lower income nation':ab,ti OR 'lower income nations':ab,ti OR 'lower income population':ab,ti OR 'lower income populations':ab,ti OR 'underserved country':ab,ti OR 'underserved countries':ab,ti OR 'underserved nation':ab,ti OR 'underserved nations':ab,ti OR 'underserved population':ab,ti OR 'underserved populations':ab,ti OR 'underserved world':ab,ti OR 'under served country':ab,ti OR 'under served countries':ab,ti OR 'under served nation':ab,ti OR 'under served nations':ab,ti OR 'under served population':ab,ti OR 'under served populations':ab,ti OR 'under served world':ab,ti OR 'deprived country':ab,ti OR 'deprived countries':ab,ti OR 'deprived nation':ab,ti OR 'deprived nations':ab,ti OR 'deprived population':ab,ti OR 'deprived populations':ab,ti OR 'deprived world':ab,ti OR 'poor country':ab,ti OR 'poor countries':ab,ti OR 'poor nation':ab,ti OR 'poor nations':ab,ti OR 'poor population':ab,ti OR 'poor populations':ab,ti OR 'poor world':ab,ti OR 'poorer country':ab,ti OR 'poorer countries':ab,ti OR 'poorer nation':ab,ti OR 'poorer nations':ab,ti OR 'poorer population':ab,ti OR 'poorer populations':ab,ti OR 'poorer world':ab,ti OR 'developing economy':ab,ti OR 'developing economies':ab,ti OR 'less developed economy':ab,ti OR 'less developed economies':ab,ti OR 'lesser developed economy':ab,ti OR 'lesser developed economies':ab,ti OR 'under developed economy':ab,ti OR 'under developed economies':ab,ti OR 'underdeveloped economy':ab,ti OR 'underdeveloped economies':ab,ti OR 'middle income economy':ab,ti OR 'middle income economies':ab,ti OR 'low income economy':ab,ti OR 'low income economies':ab,ti OR 'lower income economy':ab,ti OR 'lower income economies':ab,ti OR 'low gdp':ab,ti OR 'low gnp':ab,ti OR 'low gross domestic':ab,ti OR 'low gross national':ab,ti OR 'lower gdp':ab,ti OR 'lower gnp':ab,ti OR 'lower gross domestic':ab,ti OR 'lower gross national':ab,ti OR lmic:ab,ti OR lmics:ab,ti OR 'third world':ab,ti OR 'lami country':ab,ti OR 'lami countries':ab,ti OR 'transitional country':ab,ti OR 'transitional countries':ab,ti OR africa:ti,ab OR asia:ti,ab OR caribbean:ti,ab OR 'west indies':ti,ab OR 'south america':ti,ab OR 'latin america':ti,ab OR 'central america':ti,ab OR 'atlantic islands':ab,ti OR 'commonwealth of independent states':ab,ti OR 'pacific islands':ab,ti OR 'indian ocean islands':ab,ti OR 'eastern europe':ab,ti OR afghanistan:ti,ab OR albania:ti,ab OR algeria:ti,ab OR 'american samoa':ti,ab OR angola:ti,ab OR antigua:ti,ab OR barbuda:ti,ab OR argentina:ti,ab OR armenia:ti,ab OR armenian:ti,ab OR aruba:ti,ab OR azerbaijan:ti,ab OR bahrain:ti,ab OR bangladesh:ti,ab OR barbados:ti,ab OR benin:ti,ab OR byelarus:ti,ab OR byelorussian:ti,ab OR belarus:ti,ab OR belorussian:ti,ab OR belorussia:ti,ab OR belize:ti,ab OR bhutan:ti,ab OR bolivia:ti,ab OR bosnia:ti,ab OR herzegovina:ti,ab OR hercegovina:ti,ab OR botswana:ti,ab OR brasil:ti,ab OR brazil:ti,ab OR bulgaria:ti,ab OR 'burkina faso':ti,ab OR 'burkina fasso':ti,ab OR 'upper volta':ti,ab OR burundi:ti,ab OR urundi:ti,ab OR cambodia:ti,ab OR 'khmer republic':ti,ab OR kampuchea:ti,ab OR cameroon:ti,ab OR cameroons:ti,ab OR cameron:ti,ab OR camerons:ti,ab OR 'cape verde':ti,ab OR 'cabo verde':ti,ab OR 'central african republic':ti,ab OR chad:ti,ab OR chile:ti,ab OR china:ti,ab OR colombia:ti,ab OR comoros:ti,ab OR 'comoro islands':ti,ab OR comores:ti,ab OR mayotte:ti,ab OR congo:ti,ab OR zaire:ti,ab OR 'costa rica':ti,ab OR 'cote d`ivoire' OR 'ivory coast':ti,ab OR croatia:ti,ab OR cuba:ti,ab OR cyprus:ti,ab OR czechoslovakia:ti,ab OR 'czech republic':ti,ab OR slovakia:ti,ab OR 'slovak republic':ti,ab OR djibouti:ti,ab OR 'french somaliland':ti,ab OR dominica:ti,ab OR 'dominican republic':ti,ab OR 'east timor':ti,ab OR 'east timur':ti,ab OR 'timor leste':ti,ab OR ecuador:ti,ab OR egypt:ti,ab OR 'united arab republic':ti,ab OR 'el salvador':ti,ab OR eritrea:ti,ab OR estonia:ti,ab OR eswatini:ti,ab OR ethiopia:ti,ab OR fiji:ti,ab OR gabon:ti,ab OR 'gabonese republic':ti,ab OR gambia:ti,ab OR gaza:ti,ab OR 'georgia republic':ti,ab OR 'georgian republic':ti,ab OR ghana:ti,ab OR 'gold coast':ti,ab OR grenada:ti,ab OR guatemala:ti,ab OR guinea:ti,ab OR guam:ti,ab OR guiana:ti,ab OR guyana:ti,ab OR haiti:ti,ab OR honduras:ti,ab OR india:ti,ab OR maldives:ti,ab OR indonesia:ti,ab OR iran:ti,ab OR iraq:ti,ab OR 'isle of man':ti,ab OR jamaica:ti,ab OR jordan:ti,ab OR kazakhstan:ti,ab OR kazakh:ti,ab OR kenya:ti,ab OR kiribati:ti,ab OR korea:ti,ab OR kosovo:ti,ab OR kyrgyzstan:ti,ab OR kirghizia:ti,ab OR 'kyrgyz republic':ti,ab OR kirghiz:ti,ab OR kirgizstan:ti,ab OR 'lao pdr':ti,ab OR laos:ti,ab OR latvia:ti,ab OR lebanon:ti,ab OR lesotho:ti,ab OR basutoland:ti,ab OR liberia:ti,ab OR libya:ti,ab OR lithuania:ti,ab OR macedonia:ti,ab OR madagascar:ti,ab OR 'malagasy republic':ti,ab OR malaysia:ti,ab OR malaya:ti,ab OR malay:ti,ab OR sabah:ti,ab OR sarawak:ti,ab OR malawi:ti,ab OR nyasaland:ti,ab OR mali:ti,ab OR malta:ti,ab OR 'marshall islands':ti,ab OR mauritania:ti,ab OR mauritius:ti,ab OR melanesia:ab,ti OR 'agalega islands':ti,ab OR mexico:ti,ab OR micronesia:ti,ab OR 'middle east':ti,ab OR moldova:ti,ab OR moldovia:ti,ab OR moldovian:ti,ab OR mongolia:ti,ab OR morocco:ti,ab OR ifni:ti,ab OR mozambique:ti,ab OR myanmar:ti,ab OR myanma:ti,ab OR burma:ti,ab OR namibia:ti,ab OR nepal:ti,ab OR 'netherlands antilles':ti,ab OR 'new caledonia':ti,ab OR nicaragua:ti,ab OR niger:ti,ab OR nigeria:ti,ab OR 'northern mariana islands':ti,ab OR oman:ti,ab OR muscat:ti,ab OR pakistan:ti,ab OR palau:ti,ab OR palestine:ti,ab OR panama:ti,ab OR paraguay:ti,ab OR peru:ti,ab OR philippines:ti,ab OR philipines:ti,ab OR phillipines:ti,ab OR phillippines:ti,ab OR poland:ti,ab OR portugal:ti,ab OR 'puerto rico':ti,ab OR romania:ti,ab OR rumania:ti,ab OR roumania:ti,ab OR russia:ti,ab OR russian:ti,ab OR rwanda:ti,ab OR ruanda:ti,ab OR 'saint kitts':ti,ab OR 'st kitts':ti,ab OR nevis:ti,ab OR 'saint lucia':ti,ab OR 'st lucia':ti,ab OR 'saint vincent':ti,ab OR 'st vincent':ti,ab OR grenadines:ti,ab OR samoa:ti,ab OR 'samoan islands':ti,ab OR 'navigator island':ti,ab OR 'navigator islands':ti,ab OR 'sao tome':ti,ab OR 'saudi arabia':ti,ab OR senegal:ti,ab OR serbia:ti,ab OR montenegro:ti,ab OR seychelles:ti,ab OR 'sierra leone':ti,ab OR slovenia:ti,ab OR 'sri lanka':ti,ab OR ceylon:ti,ab OR 'solomon islands':ti,ab OR somalia:ti,ab OR sudan:ti,ab OR suriname:ti,ab OR surinam:ti,ab OR swaziland:ti,ab OR syria:ti,ab OR syrian:ti,ab OR tajikistan:ti,ab OR tadzhikistan:ti,ab OR tadjikistan:ti,ab OR tadzhik:ti,ab OR tanzania:ti,ab OR thailand:ti,ab OR togo:ti,ab OR 'togolese republic':ti,ab OR tonga:ti,ab OR trinidad:ti,ab OR tobago:ti,ab OR tunisia:ti,ab OR turkey:ti,ab OR turkmenistan:ti,ab OR turkmen:ti,ab OR tuvalu:ti,ab OR uganda:ti,ab OR ukraine:ti,ab OR uruguay:ti,ab OR ussr:ti,ab OR 'soviet union':ti,ab OR 'union of soviet socialist republics':ti,ab OR uzbekistan:ti,ab OR uzbek OR vanuatu:ti,ab OR 'new hebrides':ti,ab OR venezuela:ti,ab OR vietnam:ti,ab OR 'viet nam':ti,ab OR 'west bank':ti,ab OR yemen:ti,ab OR yugoslavia:ti,ab OR zambia:ti,ab OR zimbabwe:ti,ab OR rhodesia:ti,ab OR 'developing country'/exp OR 'africa'/de OR 'africa south of the sahara'/de OR 'north africa'/de OR 'central africa'/de OR 'asia'/de OR 'south asia'/de OR 'southeast asia'/de OR 'south america'/de OR 'central america'/de OR 'south and central america'/de OR 'atlantic islands'/de OR 'caribbean islands'/de OR 'pacific islands'/de OR 'indian ocean'/de OR 'eastern europe'/de OR 'afghanistan'/exp OR 'albania'/exp OR 'algeria'/exp OR 'american samoa'/exp OR 'angola'/exp OR 'antigua and barbuda'/exp OR 'argentina'/exp OR 'armenia'/exp OR 'azerbaijan'/exp OR 'bahrain'/exp OR 'bangladesh'/exp OR 'barbados'/exp OR 'benin'/exp OR 'belarus'/exp OR 'baltic states'/exp OR 'belize'/exp OR 'bhutan'/exp OR 'bolivia'/exp OR 'bosnia and herzegovina'/exp OR 'botswana'/exp OR 'brazil'/exp OR 'bulgaria'/exp OR 'burkina faso'/exp OR 'burundi'/exp OR 'cambodia'/exp OR 'cameroon'/exp OR 'cape verde'/exp OR 'central african republic'/exp OR 'chad'/exp OR 'chile'/exp OR 'china'/exp OR 'colombia'/exp OR 'comoros'/exp OR 'congo'/exp OR 'costa rica'/exp OR 'cote d`ivoire'/exp OR 'croatia'/exp OR 'cuba'/exp OR 'cyprus'/exp OR 'czechoslovakia'/exp OR 'czech republic'/exp OR 'slovakia'/exp OR 'djibouti'/exp OR 'democratic republic congo'/exp OR 'dominica'/exp OR 'dominican republic'/exp OR 'timor-leste'/exp OR 'ecuador'/exp OR 'egypt'/exp OR 'el salvador'/exp OR 'eritrea'/exp OR 'estonia'/exp OR 'eswatini'/exp OR 'ethiopia'/exp OR 'french guiana'/exp OR 'fiji'/exp OR 'gabon'/exp OR 'gambia'/exp OR 'georgia (republic)'/exp OR 'ghana'/exp OR 'grenada'/exp OR 'guatemala'/exp OR 'guinea'/exp OR 'guinea bissau'/exp OR 'guam'/exp OR 'guyana'/exp OR 'haiti'/exp OR 'honduras'/exp OR 'india'/exp OR 'indonesia'/exp OR 'iran'/exp OR 'iraq'/exp OR 'jamaica'/exp OR 'jordan'/exp OR 'kazakhstan'/exp OR 'kenya'/exp OR 'korea'/exp OR 'kyrgyzstan'/exp OR 'laos'/exp OR 'latvia'/exp OR 'lebanon'/exp OR 'lesotho'/exp OR 'liberia'/exp OR 'libyan arab jamahiriya'/exp OR 'lithuania'/exp OR 'macedonia (republic)'/exp OR 'madagascar'/exp OR 'malaysia'/exp OR 'malawi'/exp OR 'mali'/exp OR 'malta'/exp OR 'mauritania'/exp OR 'mauritius'/exp OR 'melanesia'/exp OR 'mexico'/exp OR 'federated states of micronesia'/exp OR 'middle east'/de OR 'moldova'/exp OR 'mongolia'/exp OR 'montenegro'/exp OR 'morocco'/exp OR 'mozambique'/exp OR 'myanmar'/exp OR 'namibia'/exp OR 'nepal'/exp OR 'netherlands antilles'/exp OR 'new caledonia'/exp OR 'nicaragua'/exp OR 'niger'/exp OR 'nigeria'/exp OR 'north korea'/exp OR 'oman'/exp OR 'pakistan'/exp OR 'palau'/exp OR 'panama'/exp OR 'papua new guinea'/exp OR 'paraguay'/exp OR 'peru'/exp OR 'philippines'/exp OR 'poland'/exp OR 'portugal'/exp OR 'puerto rico'/exp OR 'romania'/exp OR 'russian federation'/exp OR 'rwanda'/exp OR 'saint kitts and nevis'/exp OR 'saint lucia'/exp OR 'saint vincent and the grenadines'/exp OR 'samoan islands'/exp OR 'samoa'/exp OR 'saudi arabia'/exp OR 'senegal'/exp OR 'serbia'/exp OR 'montenegro (republic)'/exp OR 'seychelles'/exp OR 'sierra leone'/exp OR 'slovenia'/exp OR 'sri lanka'/exp OR 'somalia'/exp OR 'south korea'/exp OR 'south africa'/exp OR 'sudan'/exp OR 'suriname'/exp OR 'swaziland'/exp OR 'syrian arab republic'/exp OR 'tajikistan'/exp OR 'tanzania'/exp OR 'thailand'/exp OR 'togo'/exp OR 'tonga'/exp OR 'trinidad and tobago'/exp OR 'tunisia'/exp OR 'turkey (republic)'/exp OR 'turkmenistan'/exp OR 'uganda'/exp OR 'ukraine'/exp OR 'uruguay'/exp OR 'ussr'/exp OR 'uzbekistan'/exp OR 'vanuatu'/exp OR 'venezuela'/exp OR 'viet nam'/exp OR 'yemen'/exp OR 'yugoslavia'/exp OR 'yugoslavia (pre-1992)'/exp OR 'zambia'/exp OR 'zimbabwe'/exp

Scopus

( ( TITLE-ABS-KEY ( ( "Papillomavirus Vaccines" OR ( ( hpv OR "human papillomavirus" ) AND "immunization programs" ) OR "HPV vaccin*" OR "human papillomavirus vaccin*" OR "HPV immuniz*" OR "human papillomavirus immuniz*" OR "HPV immunis*" OR "human papillomavirus immunis*" ) ) AND TITLE-ABS-KEY ( "Adolescent Health Services" OR ( ( "Adolescent" OR "Child" OR adolescen* OR youth* OR "young adult*" OR "young person*" OR "young women" OR "young woman" OR teen* OR girl* OR "school age" ) AND ( "Health Services" OR "Health Education" OR "health service*" OR "healthcare" OR "health care" OR "health education" OR "preventive health" OR "promotive health" ) ) ) ) ) AND ( TITLE-ABS-KEY ( "Antigua and Barbuda " OR " Atlantic Islands " OR " Baltic States " OR " Commonwealth of Independent States " OR " Democratic People's Republic of Korea " OR " Democratic Republic of the Congo " OR " deprived countries " OR " deprived population " OR " deprived populations " OR " developing countries " OR " developing country " OR " developing economies " OR " developing economy " OR " developing nation " OR " developing nations " OR " developing population " OR " developing populations " OR " developing world " OR " Equatorial Guinea " OR " French Guiana " OR " Georgia Republic " OR " Independent State of Samoa " OR " Indian Ocean Islands " OR " lami countries " OR " lami country " OR " less developed countries " OR " less developed country " OR " less developed economies " OR " less developed economy " OR " less developed nation " OR " less developed nations " OR " less developed world " OR " lesser developed countries " OR " lesser developed nations " OR " low gdp " OR " low gnp " OR " low gross domestic " OR " low gross national " OR " low income countries " OR " low income country " OR " low income economies " OR " low income economy " OR " low income nations " OR " low income population " OR " low income populations " OR " lower gdp " OR " lower gross domestic " OR " lower income countries " OR " lower income country " OR " lower income nations " OR " lower income population " OR " lower income populations " OR " Macedonia Republic " OR " Melanesia " OR " middle income countries " OR " middle income country " OR " middle income economies " OR " middle income nation " OR " middle income nations " OR " middle income population " OR " middle income populations " OR " Pacific Islands " OR " poor countries " OR " poor country " OR " poor nation " OR " poor nations " OR " poor population " OR " poor populations " OR " poor world " OR " poorer countries " OR " poorer nations " OR " poorer population " OR " poorer populations " OR " Republic of Belarus " OR " Saint Kitts and Nevis " OR " Saint Vincent and the Grenadines " OR " South Sudan " OR " third world " OR " transitional countries " OR " transitional country " OR " Trinidad and Tobago " OR " under developed countries " OR " under developed country " OR " under developed nations " OR " under developed world " OR " under served population " OR " under served populations " OR " underdeveloped countries " OR " underdeveloped country " OR " underdeveloped economies " OR " underdeveloped nations " OR " underdeveloped population " OR " underdeveloped world " OR " underserved countries " OR " underserved nations " OR " underserved population " OR " underserved populations " OR " Afghanistan " OR " Africa " OR " Albania " OR " Algeria " OR " American Samoa " OR " Angola " OR " Argentina " OR " Armenia " OR " Asia " OR " Azerbaijan " OR " Bahrain " OR " Bangladesh " OR " Barbados " OR " Belize " OR " Benin " OR " Bhutan " OR " Bolivia " OR " Bosnia-Herzegovina " OR " Botswana " OR " Brazil " OR " Bulgaria " OR " Burkina Faso " OR " Burundi " OR " Cambodia " OR " Cameroon " OR " Cape Verde " OR " Caribbean Region " OR " Central African Republic " OR " Central America " OR " Chad " OR " Chile " OR " China " OR " Colombia " OR " Comoros " OR " Congo " OR " Costa Rica " OR " Cote d'Ivoire " OR " Croatia " OR " Cuba " OR " Cyprus " OR " Czech Republic " OR " Czechoslovakia " OR " Developing Countries " OR " Djibouti " OR " Dominica " OR " Dominican Republic " OR " East Timor " OR " Ecuador " OR " Egypt " OR " El Salvador " OR " Eritrea " OR " Estonia " OR " Ethiopia " OR " Fiji " OR " Gabon " OR " Gambia " OR " Ghana " OR " Greece " OR " Grenada " OR " Guam " OR " Guatemala " OR " Guinea " OR " Guinea-Bissau " OR " Guyana " OR " Haiti " OR " Honduras " OR " Hungary " OR " India " OR " Indonesia " OR " Iran " OR " Iraq " OR " Jamaica " OR " Jordan " OR " Kazakhstan " OR " Kenya " OR " Korea " OR " Kyrgyzstan " OR " Laos " OR " Latin America " OR " Latvia " OR " Lebanon " OR " Lesotho " OR " Liberia " OR " Libya " OR " Lithuania " OR " lmic " OR " lmics " OR " Madagascar " OR " Malawi " OR " Malaysia " OR " Mali " OR " Malta " OR " Mauritania " OR " Mauritius " OR " Mexico " OR " Micronesia " OR " Middle East " OR " Moldova " OR " Mongolia " OR " Montenegro " OR " Montenegro " OR " Morocco " OR " Mozambique " OR " Myanmar " OR " Namibia " OR " Nepal " OR " Netherlands Antilles " OR " New Caledonia " OR " Nicaragua " OR " Niger " OR " Nigeria " OR " Oman " OR " Pakistan " OR " Palau " OR " Panama " OR " Papua New Guinea " OR " Paraguay " OR " Peru " OR " Philippines " OR " Poland " OR " Portugal " OR " Puerto Rico " OR " Romania " OR " Russia " OR " Rwanda " OR " Saint Lucia " OR " Samoa " OR " Saudi Arabia " OR " Senegal " OR " Serbia " OR " Seychelles " OR " Sierra Leone " OR " Slovakia " OR " Slovenia " OR " Somalia " OR " South Africa " OR " South America " OR " Sri Lanka " OR " Sudan " OR " Suriname " OR " Swaziland " OR " Syria " OR " Tajikistan " OR " Tanzania " OR " Thailand " OR " Togo " OR " Tonga " OR " Tunisia " OR " Turkey " OR " Turkmenistan " OR " Uganda " OR " Ukraine " OR " Uruguay " OR " USSR " OR " Uzbekistan " OR " Vanuatu " OR " Venezuela " OR " Vietnam " OR " West Indies " OR " Yemen " OR " Yugoslavia " OR " Zambia " OR " Zimbabwe" ) )

Global Health (Ovid)

Global Health <1910 to 2022 Week 09>

1 exp immunization programmes/ 5472

2 (HPV or "human papillomavirus").mp. [mp=abstract, title, original title, broad terms, heading words, identifiers, cabicodes] 22361

3 1 and 2 478

4 ("Papillomavirus Vaccines" or ((HPV or "human papillomavirus") and "immunization programs") or "HPV vaccin*" or "human papillomavirus vaccin*" or "HPV immuniz*" or "human papillomavirus immuniz*" or "HPV immunis*" or "human papillomavirus immunis*").mp. [mp=abstract, title, original title, broad terms, heading words, identifiers, cabicodes] 5716

5 3 or 4 5716

6 ("Adolescent Health Services" or (("Adolescent" or "Child" or adolescen* or youth* or "young adult" or "young adults" or "young person" or "young persons" or "young people" or "young women" or "young woman" or teen* or girl* or "school age") and ("Health Services" or "Health Education" or "health service*" or "healthcare" or "health care" or "health education" or "preventive health" or "promotive health"))).mp. [mp=abstract, title, original title, broad terms, heading words, identifiers, cabicodes] 50939

7 5 and 6 1119

8 ("Antigua and Barbuda " or " Atlantic Islands " or " Baltic States " or " Commonwealth of Independent States " or " Democratic People's Republic of Korea " or " Democratic Republic of the Congo " or " deprived countries " or " deprived population " or " deprived populations " or " developing countries " or " developing country " or " developing economies " or " developing economy " or " developing nation " or " developing nations " or " developing population " or " developing populations " or " developing world " or " Equatorial Guinea " or " French Guiana " or " Georgia Republic " or " Independent State of Samoa " or " Indian Ocean Islands " or " lami countries " or " lami country " or " less developed countries " or " less developed country " or " less developed economies " or " less developed economy " or " less developed nation " or " less developed nations " or " less developed world " or " lesser developed countries " or " lesser developed nations " or " low gdp " or " low gnp " or " low gross domestic " or " low gross national " or " low income countries " or " low income country " or " low income economies " or " low income economy " or " low income nations " or " low income population " or " low income populations " or " lower gdp " or " lower gross domestic " or " lower income countries " or " lower income country " or " lower income nations " or " lower income population " or " lower income populations " or " Macedonia Republic " or " Melanesia " or " middle income countries " or " middle income country " or " middle income economies " or " middle income nation " or " middle income nations " or " middle income population " or " middle income populations " or " Pacific Islands " or " poor countries " or " poor country " or " poor nation " or " poor nations " or " poor population " or " poor populations " or " poor world " or " poorer countries " or " poorer nations " or " poorer population " or " poorer populations " or " Republic of Belarus " or " Saint Kitts and Nevis " or " Saint Vincent and the Grenadines " or " South Sudan " or " third world " or " transitional countries " or " transitional country " or " Trinidad and Tobago " or " under developed countries " or " under developed country " or " under developed nations " or " under developed world " or " under served population " or " under served populations " or " underdeveloped countries " or " underdeveloped country " or " underdeveloped economies " or " underdeveloped nations " or " underdeveloped population " or " underdeveloped world " or " underserved countries " or " underserved nations " or " underserved population " or " underserved populations " or " Afghanistan " or " Africa " or " Albania " or " Algeria " or " American Samoa " or " Angola " or " Argentina " or " Armenia " or " Asia " or " Azerbaijan " or " Bahrain " or " Bangladesh " or " Barbados " or " Belize " or " Benin " or " Bhutan " or " Bolivia " or " Bosnia-Herzegovina " or " Botswana " or " Brazil " or " Bulgaria " or " Burkina Faso " or " Burundi " or " Cambodia " or " Cameroon " or " Cape Verde " or " Caribbean Region " or " Central African Republic " or " Central America " or " Chad " or " Chile " or " China " or " Colombia " or " Comoros " or " Congo " or " Costa Rica " or " Cote d'Ivoire " or " Croatia " or " Cuba " or " Cyprus " or " Czech Republic " or " Czechoslovakia " or " Developing Countries " or " Djibouti " or " Dominica " or " Dominican Republic " or " East Timor " or " Ecuador " or " Egypt " or " El Salvador " or " Eritrea " or " Estonia " or " Ethiopia " or " Fiji " or " Gabon " or " Gambia " or " Ghana " or " Greece " or " Grenada " or " Guam " or " Guatemala " or " Guinea " or " Guinea-Bissau " or " Guyana " or " Haiti " or " Honduras " or " Hungary " or " India " or " Indonesia " or " Iran " or " Iraq " or " Jamaica " or " Jordan " or " Kazakhstan " or " Kenya " or " Korea " or " Kyrgyzstan " or " Laos " or " Latin America " or " Latvia " or " Lebanon " or " Lesotho " or " Liberia " or " Libya " or " Lithuania " or " lmic " or " lmics " or " Madagascar " or " Malawi " or " Malaysia " or " Mali " or " Malta " or " Mauritania " or " Mauritius " or " Mexico " or " Micronesia " or " Middle East " or " Moldova " or " Mongolia " or " Montenegro " or " Montenegro " or " Morocco " or " Mozambique " or " Myanmar " or " Namibia " or " Nepal " or " Netherlands Antilles " or " New Caledonia " or " Nicaragua " or " Niger " or " Nigeria " or " Oman " or " Pakistan " or " Palau " or " Panama " or " Papua New Guinea " or " Paraguay " or " Peru " or " Philippines " or " Poland " or " Portugal " or " Puerto Rico " or " Romania " or " Russia " or " Rwanda " or " Saint Lucia " or " Samoa " or " Saudi Arabia " or " Senegal " or " Serbia " or " Seychelles " or " Sierra Leone " or " Slovakia " or " Slovenia " or " Somalia " or " South Africa " or " South America " or " Sri Lanka " or " Sudan " or " Suriname " or " Swaziland " or " Syria " or " Tajikistan " or " Tanzania " or " Thailand " or " Togo " or " Tonga " or " Tunisia " or " Turkey " or " Turkmenistan " or " Uganda " or " Ukraine " or " Uruguay " or " USSR " or " Uzbekistan " or " Vanuatu " or " Venezuela " or " Vietnam " or " West Indies " or " Yemen " or " Yugoslavia " or " Zambia " or " Zimbabwe").mp. [mp=abstract, title, original title, broad terms, heading words, identifiers, cabicodes] 1504613

9 7 and 8 294

10 7 and 8 294

Global Index Medicus

https://www.globalindexmedicus.net/

Descriptor search:

"Papillomavirus Vaccines" 317 results

D20.215.894.899.498$

"Adolescent Health Services"

N02.421.044

SP2.031.232

Text word search:

( "Papillomavirus Vaccines" OR ( ( hpv OR "human papillomavirus" ) AND "immunization programs" ) OR "HPV vaccine" OR "HPV vaccines" OR "HPV vaccination" OR "HPV vaccinations" OR "human papillomavirus vaccine" OR "human papillomavirus vaccines" OR "human papillomavirus vaccination" OR "human papillomavirus vaccinations" OR "HPV immuniz*" OR "HPV immunization" OR "human papillomavirus immunizations" OR "HPV immunisation" OR "HPV immunisations" OR "human papillomavirus immunisation" OR "human papillomavirus immunisations" )

674 results

( "Adolescent Health Services" OR ( ( "Adolescent" OR "Child" OR adolescen* OR youth* OR "young adult" OR "young adults" OR "young person" OR "young persons" OR “young people” OR "young women" OR "young woman" OR teen* OR girl* OR "school age" ) AND ( "Health Services" OR "Health Education" OR "health service*" OR "healthcare" OR "health care" OR "health education" OR "preventive health" OR "promotive health" ) )

HPV vaccines and adolescents:

((mh:(D20.215.894.899.498$)) OR ( "Papillomavirus Vaccines" OR ( ( hpv OR "human papillomavirus" ) AND "immunization programs" ) OR "HPV vaccine" OR "HPV vaccines" OR "HPV vaccination" OR "HPV vaccinations" OR "human papillomavirus vaccine" OR "human papillomavirus vaccines" OR "human papillomavirus vaccination" OR "human papillomavirus vaccinations" OR "HPV immuniz*" OR "HPV immunization" OR "human papillomavirus immunizations" OR "HPV immunisation" OR "HPV immunisations" OR "human papillomavirus immunisation" OR "human papillomavirus immunisations" ) ) AND ( "Adolescent" OR "Child" OR adolescen* OR youth* OR "young adult" OR "young adults" OR "young person" OR "young persons" OR “young people” OR "young women" OR "young woman" OR teen* OR girl* OR "school age" )
