# Supplementary figures and images for: Realist review of low- to upper-middle-income country experiences on integration of HPV vaccination with other adolescent health services
Source: Vaccine. 2025 Mar 19;50:None. doi: 10.1016/j.vaccine.2025.126833 (PMC11878278; doi:10.1016/j.vaccine.2025.126833)

**Supplemental Files. Figure. Initial Proposed Theory of Change**


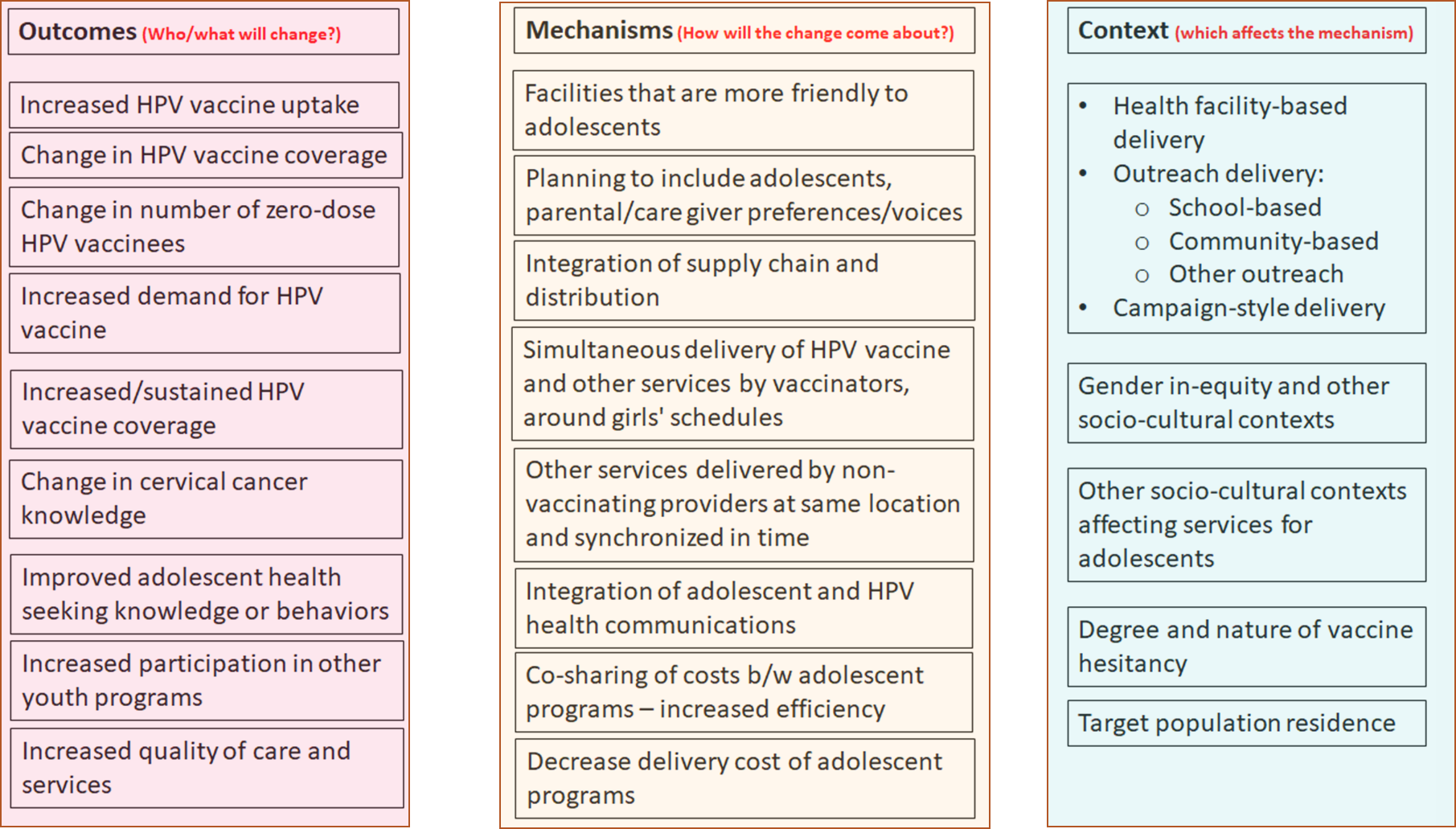

Supplement: Supplementary file 2 — Supplementary material 2 [file mmc2.docx]
